# Highly Fluorescent Distyrylnaphthalene Derivatives as a Tool for Visualization of Cellular Membranes

**DOI:** 10.3390/ma13040951

**Published:** 2020-02-20

**Authors:** Justyna Suwara, Beata Lukasik, Remigiusz Zurawinski, Roza Pawlowska, Arkadiusz Chworos

**Affiliations:** Centre of Molecular and Macromolecular Studies, Polish Academy of Sciences, Sienkiewicza 112, 90-363 Lodz, Poland; jmilczar@cbmm.lodz.pl (J.S.); blukasik@cbmm.lodz.pl (B.L.); remzur@cbmm.lodz.pl (R.Z.)

**Keywords:** distyrylnaphthalene, conjugated oligoelectrolytes, membrane stain, fluorescence imaging, cell imaging

## Abstract

Fluorescent imaging, which is an important interdisciplinary field bridging research from organic chemistry, biochemistry and cell biology has been applied for multi-dimensional detection, visualization and characterization of biological structures and processes. Especially valuable is the possibility to monitor cellular processes in real time using fluorescent probes. In this work, conjugated oligoelectrolytes and neutral derivatives with the distyrylnaphthalene core (SN-COEs) were designed, synthetized and tested for biological properties as membrane-specific fluorescent dyes for the visualization of membrane-dependent cellular processes. The group of tested compounds includes newly synthesized distyrylnaphthalene derivatives (DSNNs): a trimethylammonium derivative (DSNN-NMe_3_^+^), a phosphonate derivative (DSNN-P), a morpholine derivative (DSNN-Mor), a dihydroxyethylamine derivative (DSNN-DEA), a phosphonate potassium salt (DSNN-POK), an amino derivative (DSNN-NH_2_) and pyridinium derivative (DSNN-Py+). All compounds were tested for their biological properties, including cytotoxicity and staining efficiency towards mammalian cells. The fluorescence intensity of SN-COEs incorporated into cellular structures was analyzed by fluorescence activated cell sorting (FACS) and photoluminescence spectroscopy. The cytotoxicity results have shown that all tested SN-COEs can be safely used in the human and animal cell studies. Fluorescence and confocal microscopy observations confirm that tested COEs can be applied as fluorescent probes for the visualization of intracellular membrane components in a wide range of different cell types, including adherent and suspension cells. The staining procedure may be performed under both serum free and complete medium conditions. The presented studies have revealed the interesting biological properties of SN-COEs and confirmed their applicability as dyes for staining the membranous structures of eukaryotic cells, which may be useful for visualization of wide range of biological processes dependent of the extra-/intracellular communications and/or based on the remodeling of cellular membranes.

## 1. Introduction

In recent years, fluorescence imaging (FI) has attracted considerable attention towards their significance in life sciences, biomedicine, in diagnostics and therapy. Different types of fluorescent probes are used as biomarkers for understanding the functions and mechanisms of various biological processes from the molecular level to the cellular and tissue analysis. Both intracellular processes as well as intercellular communication may be studied using fluorescent markers. Some of the currently known and used fluorophores have some limitations, including their photostability, biocompatibility and toxicity, which limit their application potential [1]. The concerns are frequently for their sensitivity to photobleaching, low fluorescent signal intensity or narrow Stokes shift. Thus, novel photostable, non-toxic fluorescent markers with energy transfer capability and easily functionalized groups are still highly desirable. Considering these limitations, new fluorescent materials with improved properties are being developed for a variety of targets [2,3].

The most commonly used fluorescent probes for bioimaging are fluorescent proteins (FPs), organic dyes and quantum dots (QDs). Fluorescent proteins allow in fact efficient visualization of the target structure in live cells, however there are also some limitations. One of the obstacles is the need to prepare genetic constructs, which is time-consuming. Another problem is the necessity of efficient translation in tested cells and to maintain the fluorescent signal in nascent cells. Furthermore, the protein of interest in a FP-target protein construct may have different properties compared to the natural one, and what is more, the expression of the fusion gene may adversely affect cellular function. Moreover, oligomerization and aggregation of FPs can lead to a loss of function or incorrect localization of proteins. Protein aggregates may also affect the cell viability [4].

The other class of fluorescent probes is represented by fluorescent semiconductor nanocrystals (quantum dots). Quantum dots are photostable, with high quantum efficiency and usually larger Stokes shifts than organic dyes [2,5]. However, despite these advantages, the possibilities of QDs’ applications are limited by the significant toxicity caused by heavy metal components and the generation of free radicals during excitation [6]. 

Promising fluorescent probes which can overcome the above limitations seem to be conjugated polyelectrolytes (CPEs) and their shorter analogues conjugated oligoelectrolytes (COEs), which have received considerable attention since the 1990s (for a review see [7]). CPEs and COEs are specialized macromolecules with π-conjugated molecular frameworks and good light-harvesting properties [8,9]. Fluorescent materials based on this class of compounds are characterized by large absorption coefficients, wide Stokes shifts, high brightness and photostability. Due to the mentioned advantages, various applications of these compounds have been demonstrated. Their applicability in the visualization and detection of specific peptides and proteins [10], cellular imaging [11,12,13], drug tracking, gene delivery [7,14], as antimicrobial agents [15,16,17] or in bioelectrochemical systems [18] has been proven.

In searching for promising fluorescent probes for cellular applications, COEs seem to be even more attractive objects than CPEs, due to their smaller size and structural precision [19]. More attention has been paid to design the photoactivatable dyes based on COEs for in vitro and in vivo imaging of targeted cells or organelles [11,20]. One of the key examples of COEs involved in direct cellular imaging is a π-delocalized molecule, which contain the phenylenevinylene (PV-COE) framework. These distyrylbenzene and distyrylstillbene derivatives are molecules in which an intramolecular charge transfer (ICT) occurs from electron-rich dialkylamino groups to the center of conjugate. Compounds containing D-π-D structures, where D is an electron-donating group and π refers to a π-delocalized linker such as these COEs are characterized by linear and non-linear optical properties, which are valuable for uses in modern technology, including fluorescent bioimaging [21,22,23]. PV-COEs can be incorporated into synthetic and natural lipid bilayer membranes in ordered molecular orientations with superior quantum efficiency and no indication of any structure disruption [23,24]. Phenylenevinylene COEs were successfully incorporated into bacteria [15,25] and yeast cells [12,23]. Owing to their affinity for membranes and fluorophoric nature, they can quickly diffuse into mammalian cells and stain cellular membranous components, like endoplasmic reticulum and Golgi apparatuses without significant cytotoxicity [26,27]. Moreover, some date indicates that, not only intercellular membranous organelles, but also extracellular structures may be visualized using this class of compounds. One of the promising bioimaging applications of COEs is staining of the extracellular vesicles called exosomes, which are involved in cell-cell signaling. These membranous structures containing genetic material and proteins are released by cells into the extracellular environment where take part of the intercellular communication system, which is responsible for the induction of several signaling processes and pathways in neighboring cells [28]. 

One of the examined COEs-based molecular probes for direct cells staining are the 4,4′,4″-tris(4-(9,9′-bis(3′-tert-propanoatesodium) fluorenyl-2)ethynyl)phenylamine oligoelectrolytes (TEF-COONa). These star-shaped oligoelectrolytes with a triphenylamine core and negatively charged fluorenes as arms exhibit fluorescence upon accumulation in the cytoplasm and nucleus of human pancreatic carcinoma, epithelial-like (PANC-1) cells [11]. The design of organic/inorganic hybrid nanodots composed by polyhedral oligomeric silsesquioxane (POSS) and conjugated oligoelectrolyte (COE-POSS nanodots) allowed for staining the cell nucleus. The movement in the cell and the entry to the nucleus, where positively-charged COE-POSS by electrostatic attractions can form complexes with negatively-charged nucleic acids, were tracked by one-photon excited fluorescence and two-photon fluorescence imaging [29]. Knowing that nuclear modifications are related with the cancerous status of a cell, the possibility of tracking those changes is of great interest. For better bioconjugation and stability in neutral pH, complexes of COE-POSS were incorporated into chitosan/poly(ethylene glycol) nanoparticles (COE-POSS CP/PEG NPs). These biocompatible, pH-response nanoparticles were capable of triggering rapid intracellular dissolution in organelles with lower pH like endosomes (pH 5.5) and lysosomes (pH 5.0) and upon complex separation, enter the nucleus. Furthermore, surface functionalization of NPs with folate allows for the selective differentiation of cancer and normal cells based on overexpression of folate receptor in cancer cells [20]. 

A phenyl derivative, namely 4,4′-bis(4-(diphenylamino)-styryl)biphenyl (BDAVBi) was previously described as an organic compound exhibiting interesting optical and electrical properties. Because of that, BDAVBi was used as a dopant in organic light emitting diodes (OLEDs) [30,31]. This class of compounds was also applied as a photosensitive coloring material in laser digital printing [32]. Another derivative of bis(4-aminostyryl) naphthalene exhibits a pronounced fluorescence and strong affinity for fibers and for that reason it was used as an optical brightener for improving the whiteness of materials [33]. The literature reports also indicate applications of fluorescent naphthylamines and aminidonaphthalic derivatives as fluorescent dyes in cell imaging [34,35]. 

**Figure 1:**
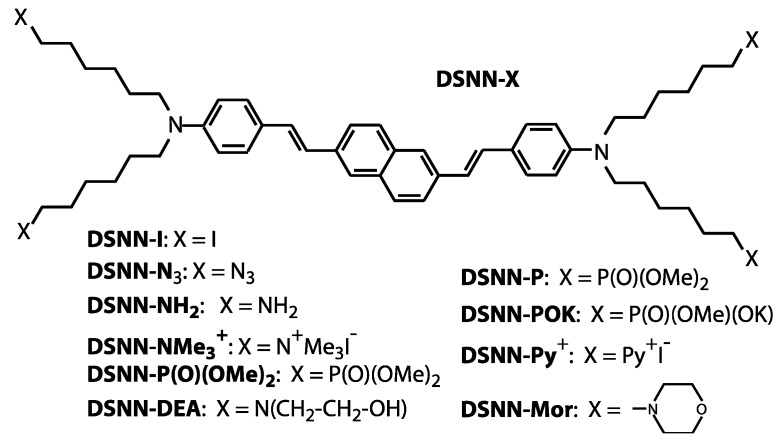
Molecular structures of the DSNN-derivatives synthetized and employed for fluorescent imaging in this study. In all presented compounds the same base core (distyrylnaphthalene) that is connected with four derivatives marked as X, where X is different depending on the compound, as shown in the scheme.

The aforementioned studies and applications clearly demonstrate that COEs constitute a propitious alternative category of fluorescent imaging materials. In our previous work, we investigated a series of phenylenevinylene COEs (PV-COEs) with excellent photostability and good cell-membrane permeability [26]. In the present work, we designed, synthetized and characterized new class of conjugated oligoelectrolytes—styrylnaphthalene COEs—as an interesting class of π-conjugated molecules for fluorescent imaging applications (Figure 1). For simplicity, we use general abbreviation SN-COEs for the whole series of analyzed distyrylnaphthalene derivatives, conjugated oligoelectrolytes and neutral derivatives. The advantage of the presented distyrylnaphthalene derivatives is that, besides high biocompatibility, upon excitation they exhibit broad emission spectra. This property can be exploited by using the green excitation available in conventional fluorescence microscopes. The optical properties of SN-COEs were investigated by absorption and fluorescence spectroscopy. The applicability of such COEs for cell membrane staining was confirmed by fluorescence microscopy. The cytotoxicity upon exposing human cell lines to these compounds was also investigated. 

## 2. Materials and Methods

### 2.1. Material Synthesis

The molecular structures of the distyrylnaphthalenes (DSNN) employed in this study are shown in Figure 1. DSNN derivatives were synthetized as previously reported [36]. The precursor compound, 2,6-bis[4-[N,N-bis(6-iodohexyl)amino]styryl]naphthalene (DSNN-I) was obtained in a four-step synthesis. Initially, a commercially available dimethyl 2,6-naphthalene dicarboxylate was reduced by lithium aluminum hydride in tetrahydrofuran at room temperature. Treatment of 2,6-bis(hydroxymethyl)naphthalene with phosphorus tribromide gave 2,6-bis(bromomethyl)- naphthalene. The next step was a Michaelis–Arbuzov reaction of the dibromide with triethyl phosphite. A key step of the synthesis was coupling a bisphosphonate with 4-(N,N-bis(6-iodohexyl)amino)benzaldehyde in a Horner-Wittig reaction. The compound DSNN-I obtained in this way was subjected to modifications within aliphatic chains according to the literature [36,37], which led to the formation of bis(aminostyryl)naphthalene derivatives. The compound DSNN-NMe_3_^+^ was synthesized by reaction of DSNN-I with excess trimethylamine in ethanol. The second cationic DSNN-Py^+^ was synthetized in the similar way, by reaction of DSNN-I with excess of trimethylamine in pyridine. The anionic derivative DSNN-POK was synthesized by the Michaelis-Arbuzov reaction of DSNN-I with trimethyl phosphite and partial hydrolysis of the dimethoxyphosphoryl group of DSNN-P in the presence of KOH. Neutral DSNN-Mor was synthesized by the reaction of DSNN-I with an excess of morpholine. A second neutral derivative DSNN-NH_2_ was prepared by the reaction of DSNN-I with sodium azide to yield DSNN-N_3_ and followed by the Staudinger reaction with triphenylphosphine to give the corresponding iminophosphorane, whose hydrolysis led to the desired compound. Another derivative, 2,6-bis(4-(N,N-bis(6-[bis(2-hydroxyethyl)-amino] hexyl) amino) styryl] naphthalene DSNN-DEA was obtained in the reaction of DSNN-I with diethanolamine in THF. The ^1^H-NMR and ^13^C-NMR spectra confirm the structure of DSNN-DEA derivative (Figure S1). Each of the obtained COEs was diluted in DMSO solution to a 5 mM concentration.

### 2.2. Cell Cultures and Reagents

All tested human and mouse cell lines were cultured in the 25 and 75 cm^2^ culture dishes in a medium appropriate for the cell type under study. Cultures were maintained in an incubator with a controlled 5% CO_2_ level and at a temperature of 37 °C. Experiments were performed with the following cell lines: human cervix carcinoma (HeLa), 293T derived from human embryonic kidney cells, human umbilical vein endothelial (HUVEC), human fibroblasts, mouse embryonic fibroblast cell line (BALB/3T3) and mouse immortalized peritoneal mesothelium (MDM) cells, suspended cells such as chronic myelogenous leukemia (K562) and human acute T lymphoblastic leukemia (MOLT4). The HeLa, K562 and MOLT-4 cells were purchased from the European Collection of Authenticated Cell Cultures (ECACC, Salisbury, UK), HUVEC cells were purchased from Life Technologies (Carlsbad, CA, USA), the human fibroblasts, BALB/3T3 and MDM cells were purchased from Celther Polska (Lodz, Poland). Cells were cultured with basic media—Dulbecco’s medium (DMEM; Sigma, St. Louis, MO, USA) (293T, fibroblasts, BALB/3T3, MDM) or in RPMI 1640 medium (Gibco, Invitrogen, Gibco, Waltham, MA, USA) (HeLa, K562, MOLT4) with addition of 10% *v*/*v* inactivated fetal bovine serum (FBS; Gibco, Invitrogen), 100 U/mL penicillin and 100 μg/mL streptomycin (Invitrogen). HUVEC cells were cultured in RPMI 1640 medium enriched in 20% *v*/*v* FBS, antibiotics (100 U/mL penicillin and 100 μg/mL streptomycin), ECGS (100 μg/mL Endothelial Cell Growth Supplement from bovine neural tissue) (Sigma-Aldrich, St. Louis, MO, USA) and heparin (10 U/mL) (Polfa S.A., Warsaw, Poland). Cell confluence was evaluated through microscopic observations. After reaching 90%–100% confluence, adherent cells (HeLa, 293T, HUVEC, fibroblasts, 3T3, MDM) were washed with Hank’s Balanced Salt Solution (HBSS; Gibco, Invitrogen) and stripped from the surface by 0.05% trypsin with EDTA solution (Gibco). K562 and MOLT4 cells were collected every 72–96 h. Before the experiments, cells were counted using a Scepter™ 2.0 Cell Counter (Merck, Saint Louis, MO, USA).

### 2.3. Cell Viability Assay

The cytotoxicity of the synthesized DSNN derivatives was assessed by the MTT (3-(4, 5-dimethylthiazol-2-yl)-2, 5-diphenyl tetrazolium bromide) test, in which the cell viability is assessed through the metabolism of soluble yellow tetrazolium salt into purple insoluble formazan. The reaction is catalyzed by mitochondrial dehydrogenase, which is active only in living cells. One day before the experiment, the cells were plated on 96-well transparent plates (Nunc) at a concentration of 10^4^ cells/well in 200 μL of fresh RPMI or DMEM medium (supplemented with 10% fetal bovine serum and 1% penicillin and streptomycin). After overnight incubation at 37 °C in a 5% CO_2_, medium was removed and replaced by fresh media containing various amount (1, 5, 10 μM) of tested compounds in DMSO. The 1 μM staurosporine was used as a reference. The final DMSO concentration in culture medium for each sample was 1%. Cell incubation was performed for 48 and 72 h in the standard conditions. After an appropriate incubation time, 25 μL of MTT (5 mg/mL) was added to each well and cells were incubated for the next 2-h to enable the reduction of MTT to purple formazan crystals. Next, the MTT containing medium was discarded and the crystals were dissolved in 100 μL of isopropanol. The plates were placed on the microplate shaker (2-h at room temperature). Then the optical density (OD) was measured spectrophotometrically by a Synergy HT 96-well plates microplate reader (Bio-Tek, Winooski, Vermont, USA) at 570 nm with a reference wavelength of 630 nm. Cell viability was determined as a percentage of living cells in the test sample relative to the non-treated control cells with 1% DMSO. Data represents the mean value from five repeats from three independent experiments. The statistical analysis (Student’s *t*-test) was done using GraphPad Prism (San Diego, CA, U.S.A.) with *p* < 0.05 (*) and *p* < 0.001 (**).

### 2.4. Visualization of Membrane Blebbing

Cells were plated on transparent 24-well plates (TPP) at density of 10^4^ cells/well in 250 μL of complete medium and left overnight at 37 °C in a 5% CO_2_ atmosphere. After overnight incubation, wells were emptied and 250 μL of fresh culture medium with 1 μM tested compounds were added. Plates were then incubated for another 24 h under standard cell culture conditions. After a 24-h incubation with tested compounds, staurosporine (0.1–10 μM) was added to the cells. Microscopic observation was made using a NisElement fluorescence microscope (Nikon, Tokyo, Japan). The conjugated oligoelectrolytes were visualized with FITC (λex = 465–495, λDM = 505, Λba = 515–555) and B-2A (long-pass, λex = 450–490, λDM = 505, λBA = 520) filter, where DM is dichroic mirror and BA is an absorption filter.

### 2.5. Optical Characterization

#### 2.5.1. Spectral Properties of SN-COEs

Samples of 5 μM solutions of DSNN-DEA were prepared in three different solvents: water, methanol and dimethyl sulfoxide (DMSO). Then, 1 mL of the tested compound solution was transferred to a quartz cuvette. Excitation and emission spectra have been recorded using a Cintra 10e UV-Visible spectrometer (GBC, Hampshire IL, USA) and a Cary Eclipse fluorescence spectrophotometer (Varian, Palo Alto, CA, USA), respectively. During emission measurements, the samples were excited by the wavelength corresponding to the maximum of absorption. 

#### 2.5.2. Optical Characterization of SN-COEs within Cellular Membranes

The cells were plated on 24-well transparent plates (TPP) at a concentration 10^4^ cells in 300 μL of complete medium (RPMI1640, 10% FBS, antibiotics) per well and incubated overnight at 37 °C in a 5% CO_2_ incubator. After overnight incubation, medium was removed and replaced by fresh medium containing 5 μM DSNN-derivatives and further incubated for 24 h in the same conditions. After that, the cells were trypsinized using 0.05% trypsin solution (Gibco) and suspended in 1 mL of PBS (Gibco). Cell suspensions were transferred to the quartz spectrophotometer cuvettes. For the excitation and emission spectra measurements, a Cintra 10e UV-Vis spectrophotometer and a Cary Eclipse fluorescence spectrophotometer were used. For the suspended cells (K562, MOLT4) trypsinization stage was not necessary.

### 2.6. Fluorescence (FL) and Confocal (CLSM) Microscopy

#### 2.6.1. Fluorescence Microscopy Imaging 

Before the planned experiment, 4 × 10^4^ cells were seeded into 24 well plates (TPP) and incubated in standard conditions. Next day, the medium was removed and replaced with 300 μL of fresh medium containing a DSNN-derivative at the final concentration 1 μM. After 24-h of incubation at 37 °C in 5% CO_2_ atmosphere, visualization was performed using a Nikon Eclipse microscope with appropriate optical filters. The conjugated oligoelectrolytes were visualized with FITC (λex = 465–495, Λdm = 505, λBA = 515–555), B-2A (long-pass, λex = 450–490, λDM = 505, λBA = 520) and UV2A filter (long-pass types, λex = 330–380, λDM = 400, λBA = 420), where DM is dichroic mirror and BA is an absorption filter. Data were recorded using NisElement (Nikon) and analyzed with ImageJ software. For fixed cells experiment, the cells were washed with PBS (Sigma Aldrich), fixed using 3.8% paraformaldehyde for 15 min in dark at room temperature and then washed three times with PBS. 

#### 2.6.2. Co-Localization Confocal Microscopy Imaging

The confocal microscopy studies were performed for the validation of intracellular localization of DSNN-derivatives. Cells were seeded on a chamber microscope slide Nunc™ Lab-Tek™ II Chamber Slide™ System (Thermo Fisher Scientific, Waltham, MA, USA at a density of 3 × 10^4^ cells per well. Next day the medium was removed and replaced by 300 μL of the appropriate culture medium containing a DSNN-derivative in the final 1 μM concentration. After 24 h of incubation, cells were treated with organelle-specific commercially available dyes. DAPI (Sigma Aldrich) was used to stain cell nuclei at a final concentration of 5 μg/mL. BODIPY^®^ TR (Life Technologies) was used to stain Golgi apparatus at a final concentration of 5 μM. MitoTracker Orange (Life Technologies, Carlsbad, CA, USA) was used to stain the mitochondria at a final concentration of 0.1 μM. The 1 μM ErTracker (Life Technologies) was used for endoplasmic reticulum imaging. After adding commercial dyes, cells were incubated for 2 h at standard conditions. Then the medium was removed and each chamber was filled with 50 μL of a 3.8% paraformaldehyde solution and incubated in dark for 15 min at room temperature. After incubation, cells were washed three times with PBS and the media chambers was removed from slides. The samples were then covered with slip using DABCO/glycerol solution and stored at 4 °C. Experiments were performed using Leica SP 5 Confocal Laser Scanning Microscopy (Leica Microsystems, Wetzlar, Germany). This setup was equipped with a HeNe laser (543 and 633 nm) and an argon laser (458, 476, 488 and 514 nm). Data were recorded using LasX software (Leica Microsystems, Wetzlar, Germany) and analyzed with ImageJ software (NIH, Bethesda, Maryland, USA).

### 2.7. Flow Cytometry Analysis

Before the experiment, 5 × 10^5^ cells were seeded into 12 well plates (TPP) and incubated under standard conditions. After overnight incubation, DSNN-derivatives at 1, 5 and 10 μM concentrations were added to the cells. After 1-h incubation, cells were centrifuged (2000RPM, 4 °C, 10 min) using a UNIVERSAL 320R centrifuge (Hettich, Tuttlingen, Germany), the pellet was then suspended in 0.5 mL of PBS and transferred to the test tubes. After that, the cells were kept on ice. Analysis was done using a FACSCalibur flow cytometer (BD Biosciences, San Jose, CA, USA) and analyzed using BD CellQuest Pro software version 6.0 Software (BD Biosciences). The statistical analysis (student’s *t*-test) was done using GraphPad Prism with *p* < 0.05 (*) and *p* < 0.001 (**).

## 3. Results and Discussion

### 3.1. Cytotoxicity Tests

**Figure 2:**
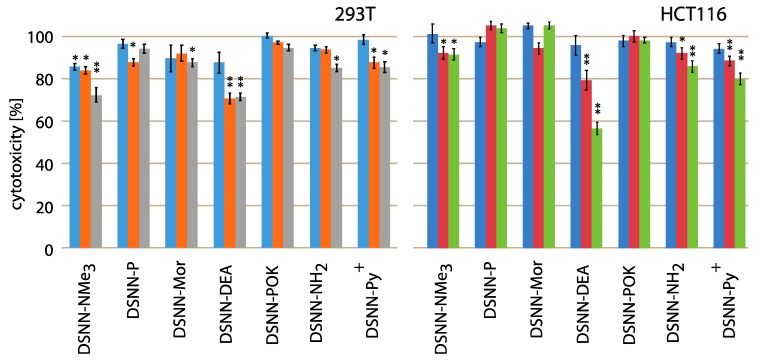
Cytotoxicity assay results (percent of living cells, vertical axis) performed with 293T (**left**) and HCT116 (**right**) adherent human cell lines after 24-h treatment with DSNN-derivatives. The blue, orange and grey bars represent viability of cells treated with DSNN-derivatives at 1, 5 and 10 μM concentrations, respectively. The experiments were done in triplicates. Results represent the mean ± SE. Statistical significance was analyzed with student’s *t*-test with *p* < 0.05 (*) and *p* < 0.001 (**).

The cytotoxicity testing of chemical compounds is one of the major requirements before biological applications, so the first step of the research was to determine the effect of the tested naphthalene derivatives on the viability of cell lines. Initially, we wanted to compare the cytotoxicity using adherent human non-cancerous cells (293T – embryonic kidney line) and adherent human cancerous cells (HCT116 – colon cancer cells line) after 24-h of concentration-dependent treatment. Relative cell survival (%) is presented as a percentage of viable treated cells relative to viable untreated cells. Data represent the average value of at least three independent experiments, each carried out in five replications. None of the tested compounds was significantly toxic for cells in tested concentration and time range (Figure 2). The slight impact on the cell viability was observed only for diethanolamine-derivative (DSNN-DEA) and DSNN-NMe_3_^+^. DSNN-DEA reduces the viability of colorectal cancer cells to 96%, 79%, 56% respectively, for 1, 5 and 10 μM concentration and the viability of 293T cells up to 70% (5 μM) and 71% (10 μM), whereas DSNN-NMe_3_^+^ at the highest concentration 10 μM showed slight cytotoxicity only for 293T cells (reduction of viability to 72%). In the case of other derivatives, the cytotoxicity effect against 293T and HCT116 cells was not observed - viability of both cell types, was maintained above 80% (Figure 2). 

Summarizing, the most of analyzed compounds had no effect on cell number or their impact was minimal, which makes them good potential fluorescent markers. The obtained toxicity results are in accordance with the literature data, which shown that the similar observation for the other classes of compounds belonging to the conjugated oligoelectrolyte family [26].

Encouraged by these results we wanted to test the toxicity effects in the presence of DSNN-derivatives in higher concentrations over longer periods of incubation—48 h (Table 1) and 72 h (Supplementary Materials, Table S1). The screening tests involved a wide range of different cell types, containing, apart from the cancers (K562, MOLT4, HCT116), also non-cancerous cell lines, both mouse (MDM and NIH/3T3) and human (293T, HUVEC and fibroblasts). Among human cancer cell lines, both suspension cells (K562, MOLT4) as well as adherent cells (HCT116) were included in these studies on the cytotoxicity of the tested compounds. 

Given the normal mouse MDM and NIH/3T3 cells, after 48-h of incubation, the DSNN-P, DSNN-Mor, DSNN-POK derivatives showed no significant effect on the cells’ survival. In the case of these derivatives, the viability of mouse cells remained at 80%–100% throughout the tested concentration range (Table 1). The greatest impact on the viability of both lines was noted for the DSNN-DEA derivative at higher concentrations. For MDM and NIH/3T3 cells, respectively, DSNN-DEA reduced the viability to 97.8% and 80.1% at a concentration of 1 μM; up to 35.3% and 36.9% at a concentration of 5 μM, and to 24.8% and 39.3% at a concentration of 10 μM. In addition, the survival rate of mouse peritoneal mesothelium cells decreased to 78.1%, 48.8%, 41.2%, respectively for 1, 5, 10 μM DSNN-NMe_3_^+^; 76%, 43.8%, 47.2% respectively for 1, 5, 10 μM DSNN-NH_2_ and up to 49%, 41.1%, 24.5% respectively for 1, 5, 10 μM DSNN-Py^+^ (Table 1). The results of assays after 72-h of incubation of MDM cells with test compounds (Supplementary Materials, Table S1) indicate the same trend of concentration-depend impact of the DSNN-NMe_3_^+^, DSNN-DEA, DSNN-NH_2_ and DSNN-Py^+^ derivatives on the cell survival. Among the tested non-cancerous human cells (293T, fibroblasts, HUVEC), the greatest effect of the tested compounds was observed in the case of human fibroblasts (Table 1, Table S1). 

The cytotoxicity analysis was also carried out using model tumor cell lines. The viability of cervical cancer cells after 48-h of incubation with the tested compounds remained above 74%. The exception was the incubation with the DSNN-DEA derivative at higher concentrations of 5 (32.6%) and 10 μM (35.9%) (Table 1). As in the case when the incubation time has been extended to 72-h, the survival of HeLa cells under the influence of DSNN-DEA decreased to 36% (5 μM) and 30.4% (10 μM), and under the influence of DSNN-NMe_3_^+^ it remained at 59.9% (5 μM) and 47.8% (10 μM) (Supplementary Materials, Table S1). Also, the viability of colorectal cancer cells (HCT116), like HeLa cells, decreased under the influence of the abovementioned trimethylammonium and dihydroxyethylamine derivatives at higher concentrations (Table 1). 

In addition to neoplastic adherent cells, cancerous suspension cells (K562 and MOLT4) were also used in the studies. The MOLT4 cell line proved to be the most sensitive cell line for the presence of tested particles. The viability of MOLT4 cells after 72-h of incubation decreased to 7.8% and 7% (respectively for 5 and 10 μM DSNN-NMe_3_^+^); up to 6.9% and 3.6% (for 5 and 10 μM DSNN-DEA); to 7.6% and 5.3% (for 5 and 10 μM DSNN-Py^+^) (Supplementary Materials, Table S1). The presented results show in most cases concentration-dependent decrease of cell viability. However, the MTT assays clearly confirm that the selected DSNN derivatives (DSNN-P, DSNN-Mor, DSNN-DEA) could be added at 1 μM concentration to most mammalian cells without significant (>5%) toxic effects. Such a concentration is sufficient to observe the fluorescent signal; thus, cytotoxicity results confirm the applicability of these compounds in fluorescent imaging in wild range of human cell types, both cancer and non-cancerous, adherent and suspension and also other mammalian (mouse) cells. 

### 3.2. Evaluation of Spectral Properties of the DSNN-Derivatives

The optical properties of tested compounds (their absorption and emission spectra in three solvents of different polarity) were appraised in previous work [36]. The spectral characteristics of the new developed dihydroxyethylamino derivative are presented in the Supplementary Materials (Figure S2). In these studies, we study the suitability of the described compounds for applications as fluorescent markers in biological systems, thus it was important to determine the fluorescence emission profiles of the DSNN-derivatives after incorporation into intracellular membranous structures. For this purpose, suspended human leukemia cells line (K562) and adherent cervical cancer cells (HeLa) were used. Cells after 24-h incubation with 5 μM DSNN-derivatives were centrifuged and suspended in PBS solution. As a reference, 5 μM solutions of test compounds in PBS were applied.

**Figure 3:**
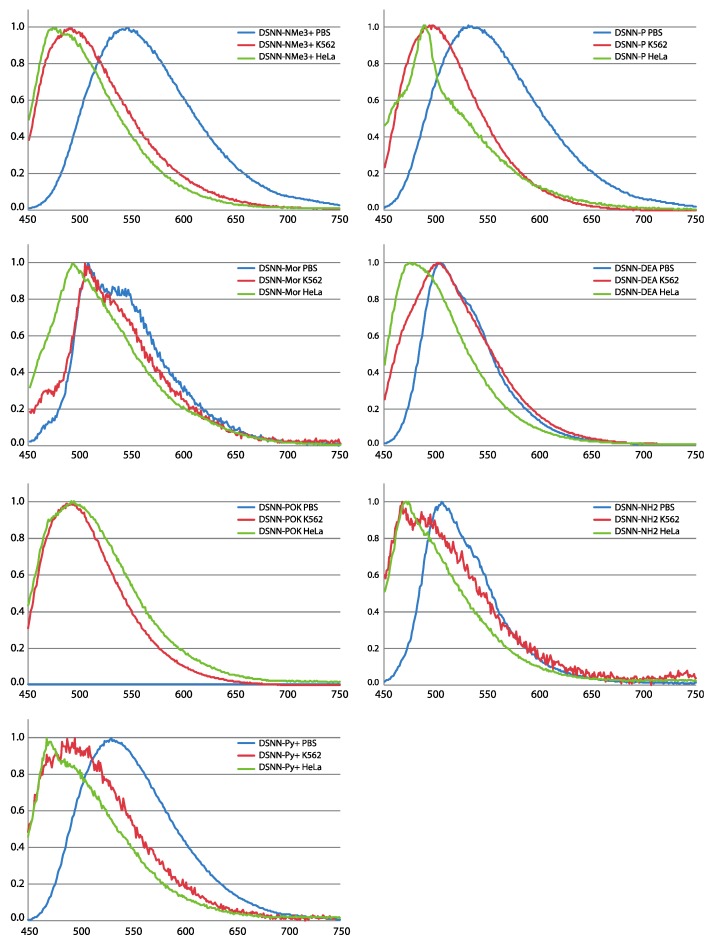
Normalized emission spectra (PL) of tested compounds: DSNN-NMe_3_^+^, DSNN-P, DSNN-Mor, DSNN-DEA, DSNN-POK, DSNN-NH_2_, DSNN-Py^+^ in PBS and after incorporation of compounds into K562 and HeLa cells. The experiments were done in triplicate.

The spectral analysis provides information on how the optical characteristics of tested compounds change upon incorporation into membranous cell structures (Figure 3). For most of tested DSNN-derivatives, except of DSNN-POK, a hypsochromic shift of λ_em_ was observed for chromophores associated with the cell membranes compared to those dissolved in a PBS solution. Among tested SN-COEs, the largest λ_em_ shifts were observed for DSNN-NMe_3_^+^. The maximum emission λ_em_ of DSNN-NMe_3_^+^ in PBS is 546 nm, in K562 cells it is shifted to 489 nm, in HeLa cells up to 473 nm. The smallest blue shift was observed for morpholine derivative: 3 and 15 nm in K562 and HeLa cells, respectively. In the case of the DSNN-POK derivative, the emission intensity in PBS was relatively low (blue line in Figure S2). It can be related with the fact that conjugated part of highly soluble phosphates is flexible in aqueous solutions, while stiffened molecules inside of lipid membrane recover electron transfer and fluorescence efficiency (λ_em_ of DSNN-POK is 492 nm for both, incorporated in K562 and HeLa cells).

Summarizing, all tested SN-COEs show a blue-shift from aqueous PBS buffer to suspended K562 cells and to HeLa, suggesting an increase in hydrophobicity. This phenomenon was previously observed for SN-COEs diluted in organic solvents [36] and for previously tested phenylene-vinylene derivatives (PV-COEs) [23,27]. Moreover, the higher fluorescence intensity of the tested compounds after incorporation into lipid membranes is depending on their structure. For distyrylstilbene (DSSN^+^) and distyrylbenzene (DSBN^+^) oligoelectrolytes orientation within lipid bilayers was previously described [23]. Based on that, the new conjugated oligoelectrolytes were designed to have the hydrophobic core part in the inner part of the membrane and the ionic pendant groups directed outside [23,36,38]. The accurate mechanism of incorporation of COE into membrane is still not clear, however it was recently shown, that COEs may cause the membrane deformation through the lipid phosphate groups leaning toward the center of the bilayer by COE side chains [39,40,41].

### 3.3. Imaging of Cells Labeled by DSNN-Derivatives

**Figure 4:**
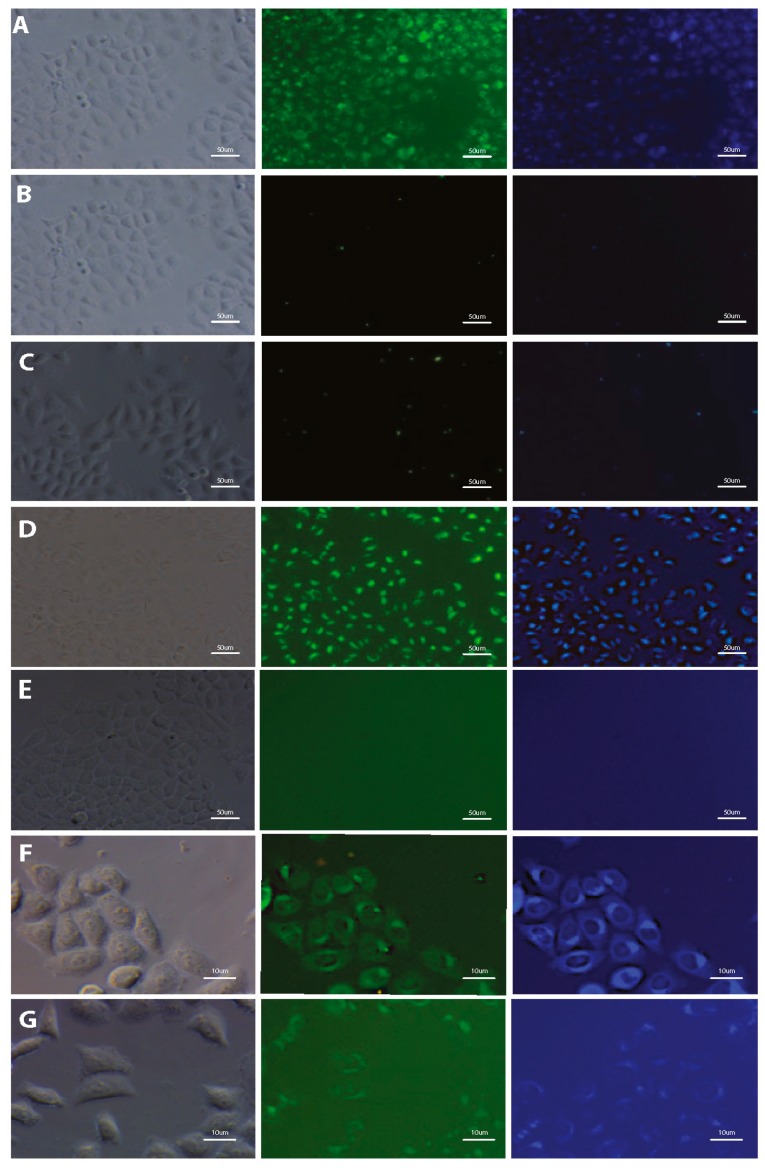
Fluorescence microscopy images of HeLa cells after 24-h incubation with tested SN-COEs at 1 μM concentration. (**A**) DSNN-NMe_3_^+^, (**B**) DSNN-P, (**C**) DSNN-Mor, (**D**) DSNN-DEA, (**E**) DSNN-POK, (**F**) DSNN-NH_2_, (**G**) DSNN-Py**^+^**. Images A–E are collected at 20× magnification, images F–G at 40×. Left panel—phase contrasts, middle panel—B2A filter (exposure time—A 1 s, B 4 s, C 1 s, D 1 s, E 1 s, F 1 s, G 3 s), right panel—UV-2A filter (A–E 200 ms) and DAPI (F–G 1 s).

The potential of using styrylnaphtalene based compounds (SN-COEs) as fluorescent probes, was verified by staining experiments using fluorescent and confocal microscopy techniques. Initial microscopic observation for living HeLa cells after 24-h incubation with 1 μM compounds showed effective cell staining using DSNN-NMe_3_^+^, DSNN-DEA, DSNN-NH_2_ and DSNN-Py^+^ derivatives (Figure 4A,D,F,G, respectively). However, in the same observation parameters for all cases, weak intracellular fluorescence was detected for DSNN-P, DSNN-Mor and DSNN-POK derivatives (Figure 4B,C,E, respectively). Interestingly, the emission spectra of these SN-COEs (Figure 3) indicate the presence of fluorescent signal from the compounds incorporated into the cell membranes (λ_em_ shift), however, their intensity is probably too low for detection using optical microscopy techniques. In case of phosphonate derivatives, the reason for the weak fluorescence may be the strong repulsive forces between negative phosphonate groups from DSNN derivatives and membranes. 

Based on the microscopic studies, two best candidates—DSNN-DEA and DSNN-NMe_3_^+^—were selected among the tested SN-COEs. The most promising compounds were then used for further analysis including subsequent tests confirming their usefulness for cellular labeling.

**Figure 5:**
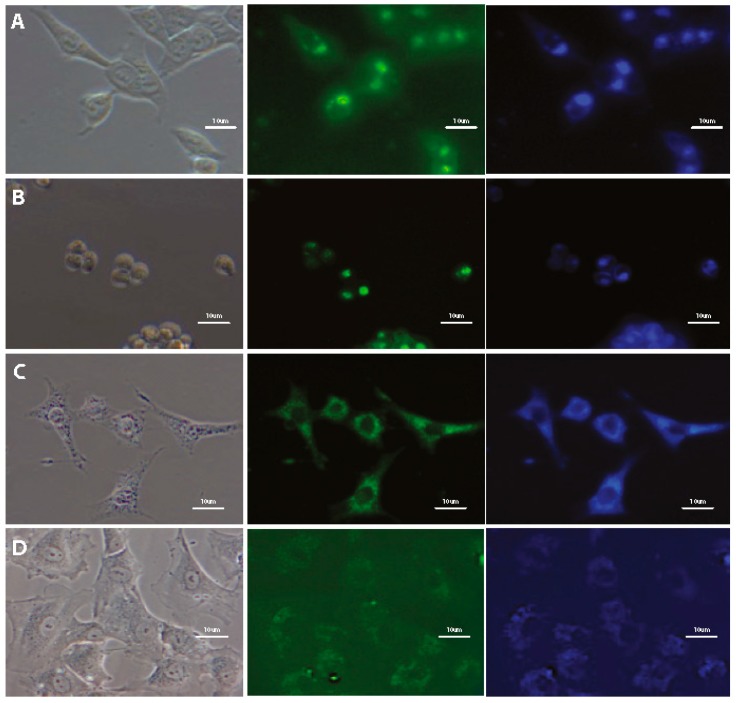
Fluorescence microscopy images of different mammalian cells after 24-h incubation with tested SN-COEs at 1 μM concentration, collected at 40× magnification. Left panel—phase contrasts, middle panel—B2A filter, right panel—UV-2A filter. (**A**) Live HCT116 cells with 1 μM DSNN-DEA (B2A 3 s, DAPI 3 s); (**B**) Live MOLT4 cells with 1 μM DSNN-DEA (FITC 2s, DAPI 1s); (**C**) Fixed NIH/3T3 cells with 1 μM DSNN-NMe_3_^+^ (B2A 2 s, DAPI 1 s); (**D**) Fixed HUVEC cells with DSNN-NMe_3_^+^ (B2A 1 s, DAPI 1 s).

Initially, the applicability of selected DSNN-derivatives for cell staining of various types of mammalian cells was tested. A wide range of different cell types was used for the study. Research included both, adherent (HeLa, HCT116, 293T, HUVEC,) and suspended (MOLT4, K562) cell types, as well as cancerous and non-cancerous cells (HUVEC, 293T, fibroblasts). Tested compounds stain different types of analyzed human cells (Figure 5), which showed great universality of these fluorescent markers. Besides human cells, two types of mouse cells (NIH/3T3 and MDM) were also analyzed and in these models similar staining profile were observed (Figure 5C). Furthermore, the cell staining profiles by SN-COEs under various cell culture conditions were also determined and compared. A series of experiments carried out on HeLa cells with 1 μM SN-COEs in different variants of cell culture media (HBSS as a poor medium, RPMI1640 as a basic medium and RPMI1640 with 10% addition of FBS and antibiotics as a complete medium) do not indicate the differences in the cell staining profiles depending on culture conditions (Supplementary Materials, Figure S3).

The obtained data confirm the applicability of the tested compounds for dyeing of membranous structures under various experimental conditions. That extends the range of their possible applications beyond the processes requiring specific experimental procedures like cell fixing, and washing. Hereby, it has been confirmed that staining of cell membranes with SN-COEs may be performed in complete culture medium containing fetal bovine serum without the need for medium exchange. Moreover, the results indicate, that the presence of antibiotics in the culture medium also do not interfere with the fluorescent signal. Such data provide valuable information needed to design future SN-COE research. With such a wide range of possible experimental conditions, these compounds may also be used for monitoring of serum-free sensitive processes. 

In addition, both, the possibility of using compounds for real-time imaging as well as their usefulness in the case of fixed microscopic preparations was assessed. Comparison of fixed and non-fixed staining was performed to examine the applicability of tested compounds under different conditions, with various procedural requirements. Obtained data shown, that the fluorescence emission in live (Figure 4) and fixed (Figure S5) HeLa cells emanate from similar intracellular compartments, so it can be concluded that the process of cell fixation using 3.8% paraformaldehyde does not change significantly the staining profile of the cells. Thus, it will be possible to use COE in colocalization staining requiring cell fixation. The possibility of using these compounds in e.g., immunocytochemistry, significantly extends its applicability to study key cellular processes. 

On the other hand, the ability to stain live cells enables tracking of cellular processes in real time, which is especially important for monitoring changes occurring with membrane rearrangement or remodeling of cell-cell junctions. Absence of background fluorescent signal from COEs diluted in culture medium facilitate their use by eliminating washing steps. In consequence, it is possible to visualize, invisible in phase contrast, structures such as membrane blebs (Figure S4). This advantage significantly extends potential application of tested COEs and enables visualization such processes as e.g., filopodia formation or plasma membrane blebbing. Thus, it is possible to track the changes associated with cell motility, intercellular communication, apoptosis or epithelial-mesenchymal transition (EMT). 

### 3.4. Subcellular Localization

**Figure 6:**
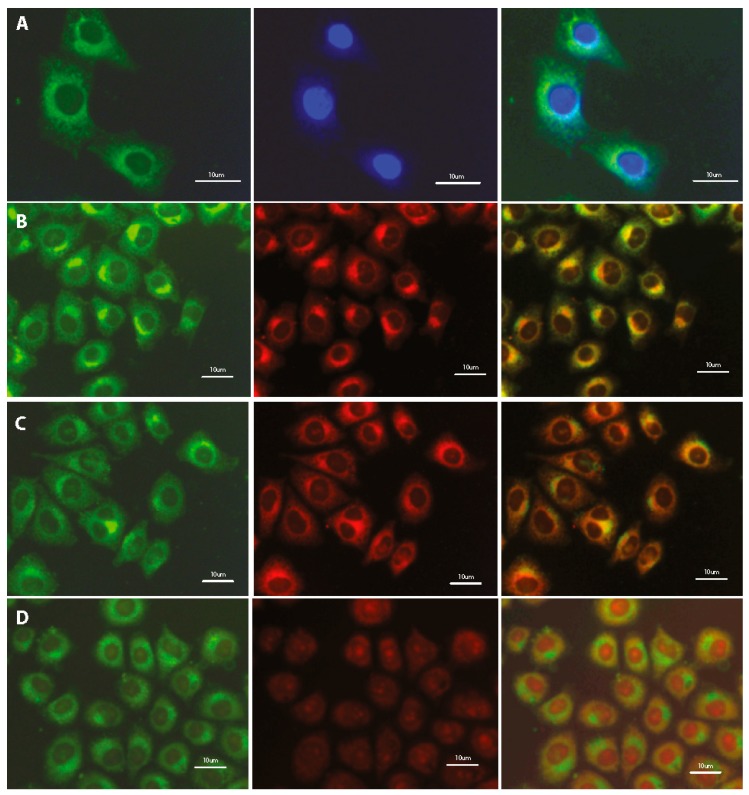
Fluorescence microscopy images of fixed HeLa cells after 24-h incubation with 1 μM DSNN-NMe_3_^+^ co-labeled with (**A**) 5 μg/mL DAPI; (**B**) 5 μM BODIPY^®^ TR; (**C**) 1 μM ErTracker; (**D**) 0.1 μM MitoTracker Orange. Left panel—B2A filter (exposure time—A 2 s, B 3 s, C 4 s, D 3 s); middle panel—DAPI filter (A 1 s) or Texas red filter (B 4 s, C 4 s, D 3 s); right panel—merged of left and middle panels. Commercial fluorescent probes were used in the concentration recommended by manufacturer.

**Figure 7:**
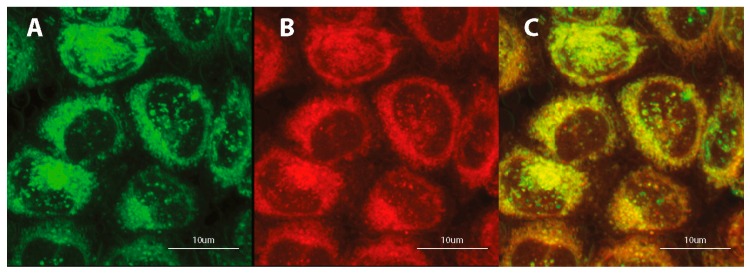
Confocal microscopy images of fixed HeLa cells after 24-h incubation with 1 μM DSNN-NMe_3_^+^ at 1 μM concentration, co-labeled 2-h with 1 μM ErTracker. Left panel A— green fluorescence of tested compounds, B — red fluorescence of ErTracker, C — merged of left and middle panels.

Preliminary studies indicate the localization of tested fluorescent dyes in the cell membrane, endoplasmic reticulum and Golgi apparatus, without staining the nucleus. Additional treatment with fluorescent probes, which are specific for staining individual intracellular structures allowed for confirmation the staining specification. Double staining with the COE and well-known marker for adenine-thymine rich region in DNA (DAPI) confirms the absence of tested compounds inside the cell nucleus (Figure 6A, Figure S5A). Further co-localization tests indicate the fluorescence emission in intracellular membrane-rich organelles such as Golgi apparatus (Figure 6B, Figure S5B) and endoplasmic reticulum (Figure 6C; Figure S5C; Figure 7) but not mitochondria (Figure 6D, Figures S5D and S6).

Summarizing, the fluorescence and confocal microscopic observations allow us to conclude, that some of tested compounds from the SN-COEs class effectively stain intracellular membranous structures like endoplasmic reticulum and Golgi apparatus, but do not mark the nucleus and mitochondria. 

Noteworthy is also that, in contrast to presented SN-COEs, the commonly used commercial dyes exhibit the fluorescent signal in culture medium. Moreover, most of them have limited selectivity for staining structures, as well as their working concentration needed for effective cell staining is higher than for SN-COEs.

Cell membranes and membranous structures are difficult objects for research due to their morphologies and dynamic remodeling, therefore, new markers enabling examination of membrane-related processes are still desired [42]. Among the compounds used for this purpose are small molecules, like naphthalene derivatives [43], styrylpyridines [44,45], peryleneimido diesters [46] or BODIPY-based particles [47]. Although some of these can be used in similar or lower concentrations and also do not show significant cytotoxicity, nevertheless often exhibit other limitation e.g., low signal to noise ratio or requirement of washout.

### 3.5. Quantification of Cellular Uptake by Flow Cytometry

**Figure 8:**
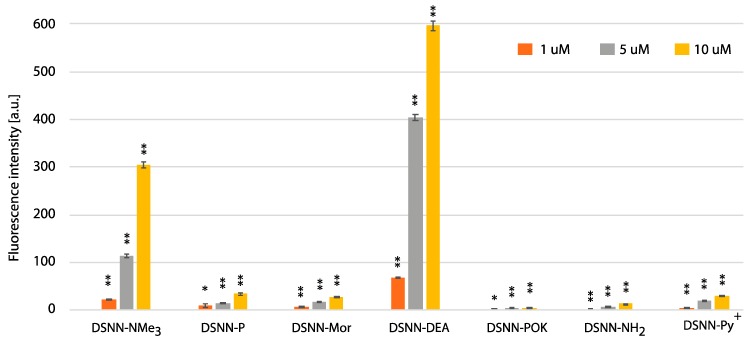
The flow cytometry analysis - fluorescence intensity of the tested compounds inside K562 cells. Data were collected after one-hour incubation of the cells in full medium. The experiments were done in triplicate. Bars represent the average +/- SE. Statistical significance was analyzed with student’s *t*-test with *p* < 0.05 (*) and *p* < 0.001 (**).

**Figure 9:**
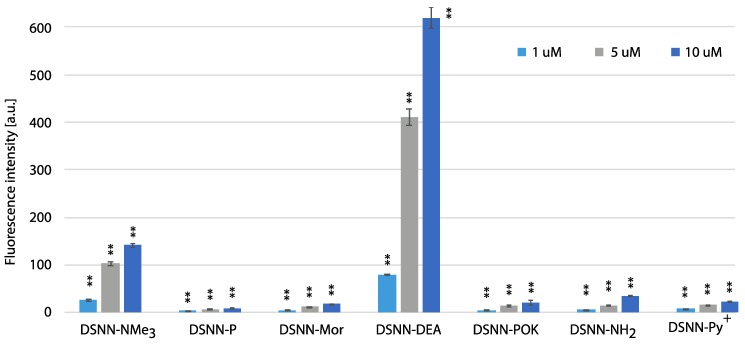
The flow cytometry analysis - fluorescence intensity of the tested compounds inside MOLT4 cells. Data were collected after one-hour incubation of the cells in full medium. The experiments were done in triplicate. Bars represent the average +/- SE. Statistical significance was analyzed with student’s *t*-test with *p* < 0.05 (*) and *p* < 0.001 (**).

In order to estimate the yield of SN-COEs cellular uptake, the intracellular fluorescence intensity was done using the flow cytometry Fluorescence Activated Cell Sorting (FACS) method, which allows for sorting cells based on their size, granularity and fluorescence detection. The fluorescence signal was measured for SN-COEs after their incorporation into cellular membranes, with the rejection of non-specific signals from small structures, fragments of membrane, large aggregates, etc. For these tests, human leukemia cells—K562 (Figure 8, Figure S7) and MOLT4 (Figure 9, Figure S8)—were selected. Performed analysis showed the dependence of the fluorescence intensity on the concentration of the tested compounds. Concentration-dependent increase in fluorescence intensity was observed for all tested COEs. 

The highest level of fluorescence intensity was measured for DSNN-NMe_3_^+^ and DSNN-DEA derivatives in both cell lines. In K562 cells, the fluorescence intensity level of the DSNN-NMe_3_^+^ derivative was 24, 114, 305a.u.; while for the DSNN-DEA derivative it was 69, 403, 596a.u. (for 1, 5 and 10 μM respectively). In MOLT4 cells, the fluorescence intensity level for the DSNN-NMe_3_^+^ derivative was 26, 103, 140a.u.; and for the DSNN-DEA derivative it was 79, 409, 617a.u. (for 1, 5 and 10 μM respectively). The fluorescence intensity of the other derivatives was relatively low, and that is in good agreement with microscopic observations. Illustrative scatterplots from readings from the forward detector (FCS) (5.9) versus fluorescence readings (FL1 488 nm) and the corresponding histograms for K562 and MOLT4 cells stained with tested SN-COEs in the concentration range 1–10 μM are available in Supplementary Materials. 

## 4. Conclusions

In the presented work, a set of conjugated oligoelectrolytes and neutral derivatives with a styrylonaphtalene core (SN-COEs) was tested for staining of membranous organelles of mammalian cells. Our studies include spectrometric characterization of DSNN-derivatives and assessment of impact on the viability of model cell lines, which allowed for determination of their biocompatibility, cytotoxicity and the stability in biological cellular system. In addition, intracellular staining allowed determination of cell staining profiles of tested compounds and the evaluation of their potential for flow cytometry analysis.

The comparison of all tested SN-COEs exhibits their similar optical properties and ability for incorporation into the lipid membrane with two amino derivatives (trimetylamino and dihydroxyethylamino) to be the best. In addition, the intercalation of all tested compounds to natural lipid bilayers significantly increases the fluorescent signal. Furthermore, analysis of the impact of DSNN-derivatives on the viability of different types of mammalian cells indicates the possibility of safe use in the cells in their effective for staining micromolar concentration. In-depth microscopic analysis (fluorescence and confocal microscopy) confirmed the specific incorporation of tested compounds into the cell membranes and intracellular membranous organelles. Besides no presence of SN-COEs in the cell nucleus was showed in co-localization studies. The usefulness of these fluorescent probes for observations both, life as well as fixed cells has also been confirmed. Moreover, some of tested derivatives enter into the cells quickly, what allows for observation of intracellular fluorescence after just a few minutes of incubation. The specificity in the labeling of membranous structures by the tested compounds opens the way to the study of cellular processes such as epithelial-mesenchymal transition, cell motility or formation of apoptotic blebs during programmed cell death. In addition to imaging cells or tissues, these compounds can be successfully used in cytometry techniques. 

## Figures and Tables

**Table 1:** In vitro cytotoxicity assay results (% of living cells) performed on various cell lines after 48 h of treatment with DSNN-compounds in 1, 5 and 10 μM. The experiments were done in triplicate. The results represent the mean ± standard error [%].

Compound	Concentration [μM]	Cytotoxicity in Individual Cell Lines [%]					
		MDM	NIH/3T3	293T	Fibroblasts	HCT116	K562
DSNN-NMe_3_^+^	1	78.1 ± 0.6	96.3 ± 3	95.5 ± 2.0	56.0 ± 2.8	91.9 ± 2.8	78.2 ± 2.1
	5	48.9 ± 2.8	73.0 ± 2.1	85.1 ± 3.3	54.6 ± 8.5	57.5 ± 2.4	73.7 ± 1.0
	10	41.2 ± 2.1	57.1 ± 2.3	77.4 ± 3.5	47.2 ± 8.8	44.2 ± 1.8	64.3 ± 6.2
DSNN-P	1	111.4 ± 3.0	98.2 ± 2.6	105.7 ± 3.1	83.0 ± 6.9	109.0 ± 1.8	96.6 ± 5.2
	5	105.8 ± 1.7	95.5 ± 3.9	101.8 ± 3.6	90.4 ± 3.2	112.7 ± 1.8	93.1 ± 1.0
	10	110.5 ± 2.6	89.6 ± 2.4	103.7 ± 3.6	97.6 ± 5.7	104.6 ± 2.4	89.6 ± 7.9
DSNN-Mor	1	110.6 ± 1.5	96.4 ± 3.1	99.7 ± 3.3	96.9 ± 5.7	95.3 ± 2.0	98.7 ± 0.3
	5	104.2 ± 2.9	99.5 ± 1.4	104.8 ± 3.0	106.1 ± 4.6	116.1 ± 3.5	94.4 ± 1.3
	10	115.0 ± 5.3	95.0 ± 3.7	99.9 ± 3.2	99.3 ± 7.0	90.6 ± 2.8	78.8 ± 0.8
DSNN-DEA	1	97.8 ± 1.9	80.1 ± 1.8	98.0 ± 2.7	86.4 ± 6.7	107.1 ± 1.7	101.9 ± 2.4
	5	35.3 ± 3.6	36.9 ± 2.3	81.0 ± 2.8	94.6 ± 12.9	73.1 ± 1.7	54.0 ± 2.3
	10	24.7 ± 3.1	39.3 ± 5.4	72.0 ± 4.7	59.9 ± 5.9	20.4 ± 1.8	49.2 ± 0.8
DSNN-POK	1	86.0 ± 2.2	99.1 ± 4.6	102.5 ± 2.1	149.4 ± 4.6	102.6 ± 3.0	90.8 ± 0.7
	5	89.8 ± 5.4	100.3 ± 3.8	104.4 ± 3.8	146.0 ± 9.7	101.2 ± 6.4	85.0 ± 2.4
	10	81.7 ± 2.8	90.0 ± 3.4	109.0 ± 3.5	111.2 ± 6.4	82.6 ± 2.4	85.6 ± 0.4
DSNN-NH_2_	1	76.0 ± 1.9	94.8 ± 3.5	96.9 ± 3.3	118.8 ± 4.7	95.5 ± 3.1	102.8 ± 5.0
	5	43.8 ± 0.9	70.7 ± 2.8	94.9 ± 2.5	87.8 ± 5.0	72.5 ± 2.2	107.8 ± 3.5
	10	47.2 ± 3.2	59.8 ± 3.0	92.2 ± 2.2	80.1 ± 6.2	48.0 ± 2.6	107.4 ± 0.4
DSNN-Py^+^	1	49.0 ± 0.6	80.6 ± 3.0	96.8 ± 3.3	117.2 ± 6.4	81.0 ± 3.4	108.6 ± 5.8
	5	41.1 ± 3.8	68.4 ± 2.0	91.1 ± 2.6	117.5 ± 9.5	51.7 ± 2.7	78.7 ± 2.6
	10	24.5 ± 1.5	52.6 ± 2.7	84.0 ± 2.2	91.7 ± 9.9	38.4 ± 2.3	59.5 ± 2.3

## Supplementary Materials

The following are available online at https://www.mdpi.com/1996-1944/13/4/951/s1, Figure S1: NMR spectra, Figure S2: Absorption and emission spectra of DSNN-DEA, Figure S3–Figure S6: Fluorescence and confocal microscopy images, Figure S7–Figure S8: FACS data, Table S1: Cytotoxicity results.

## References

[1] Specht E.A., Braselmann E., Palmer A.E. (2017). A Critical and Comparative Review of Fluorescent Tools for Live-Cell Imaging. Annu. Rev. Physiol..

[2] Li K., Liu B. (2012). Polymer encapsulated conjugated polymer nanoparticles for fluorescence bioimaging. J. Mater. Chem..

[3] Chen K., Chen X. (2010). Design and development of molecular imaging probes. Curr. Top. Med. Chem..

[4] Wiedenmann J., Oswald F., Nienhaus G.U. (2009). Fluorescent proteins for live cell imaging: Opportunities, limitations, and challenges. IUBMB Life.

[5] Michalet X., Pinaud F.F., Bentolila L.A., Tsay J.M., Doose S., Li J.J., Sundaresan G., Wu A.M., Gambhir S.S., Weiss S. (2005). Quantum dots for live cells, in vivo imaging, and diagnostics. Science.

[6] Hardman R. (2006). A toxicologic review of quantum dots: Toxicity depends on physicochemical and environmental factors. Environ. Health Perspect..

[7] Feng X.L., Lv F.T., Liu L.B., Yang Q., Wang S., Bazan G.C. (2012). A Highly Emissive Conjugated Polyelectrolyte Vector for Gene Delivery and Transfection. Adv. Mater..

[8] Wang V.B., Du J., Chen X.F., Thomas A.W., Kirchhofer N.D., Garner L.E., Maw M.T., Poh W.H., Hinks J., Wuertz S. (2013). Improving charge collection in Escherichia coli-carbon electrode devices with conjugated oligoelectrolytes. Phys. Chem. Chem. Phys..

[9] Lee Y., Yang I., Lee J.E., Hwang S., Lee J.W., Um S.S., Nguyen T.L., Yoo P.J., Woo H.Y., Park J. (2013). Enhanced Photocurrent Generation by Forster Resonance Energy Transfer between Phospholipid-Assembled Conjugated Oligoelectrolytes and Nile Red. J. Phys. Chem. C.

[10] Hammarstrom P., Simon R., Nystrom S., Konradsson P., Aslund A., Nilsson K.P.R. (2010). A Fluorescent Pentameric Thiophene Derivative Detects in Vitro-Formed Prefibrillar Protein Aggregates. Biochemistry.

[11] Song W.L., Jiang R.C., Yuan Y., Lu X.M., Hu W.B., Fan Q.L., Huang W. (2013). Star-shaped conjugated oligoelectrolyte for bioimaging in living cells. Chin. Sci. Bull..

[12] Thomas A.W., Henson Z.B., Du J., Vandenberg C.A., Bazan G.C. (2014). Synthesis, Characterization, and Biological Affinity of a Near-Infrared-Emitting Conjugated Oligoelectrolyte. J. Am. Chem. Soc..

[13] Woo S.J., Park S., Jeong J.E., Hong Y., Ku M., Kim B.Y., Jang I.H., Heo S.C., Wang T., Kim K.H. (2016). Synthesis and Characterization of Water-Soluble Conjugated Oligoelectrolytes for Near-Infrared Fluorescence Biological Imaging. ACS Appl. Mater. Interfaces.

[14] Wang G., Yin H., Ng J.C.Y., Cai L.P., Li J., Tang B.Z., Liu B. (2013). Polyethyleneimine-grafted hyperbranched conjugated polyelectrolytes: Synthesis and imaging of gene delivery. Polym. Chem..

[15] Yan H., Rengert Z.D., Thomas A.W., Rehermann C., Hinks J., Bazan G.C. (2016). Influence of molecular structure on the antimicrobial function of phenylenevinylene conjugated oligoelectrolytes. Chem. Sci..

[16] Wang B., Feng G., Seifrid M., Wang M., Liu B., Bazan G.C. (2017). Antibacterial Narrow-Band-Gap Conjugated Oligoelectrolytes with High Photothermal Conversion Efficiency. Angew. Chem. Int. Ed. Engl..

[17] Zhou C., Chia G.W.N., Ho J.C.S., Seviour T., Sailov T., Liedberg B., Kjelleberg S., Hinks J., Bazan G.C. (2018). Informed Molecular Design of Conjugated Oligoelectrolytes To Increase Cell Affinity and Antimicrobial Activity. Angew. Chem. Int. Ed. Engl..

[18] Yan H., Catania C., Bazan G.C. (2015). Membrane-intercalating conjugated oligoelectrolytes: Impact on bioelectrochemical systems. Adv. Mater..

[19] Ortony J.H., Chatterjee T., Garner L.E., Chworos A., Mikhailovsky A., Kramer E.J., Bazan G.C. (2011). Self-assembly of an optically active conjugated oligoelectrolyte. J. Am. Chem. Soc..

[20] Ding D., Pu K.Y., Li K., Liu B. (2011). Conjugated oligoelectrolyte-polyhedral oligomeric silsesquioxane loaded pH-responsive nanoparticles for targeted fluorescence imaging of cancer cell nucleus. Chem. Commun..

[21] Woo H.Y., Korystov D., Mikhailovsky A., Nguyen T.Q., Bazan G.C. (2005). Two-photon absorption in aqueous micellar solutions. J. Am. Chem. Soc..

[22] Woo H.Y., Liu B., Kohler B., Korystov D., Mikhailovsky A., Bazan G.C. (2005). Solvent effects on the two-photon absorption of distyrylbenzene chromophores. J. Am. Chem. Soc..

[23] Garner L.E., Park J., Dyar S.M., Chworos A., Sumner J.J., Bazan G.C. (2010). Modification of the optoelectronic properties of membranes via insertion of amphiphilic phenylenevinylene oligoelectrolytes. J. Am. Chem. Soc..

[24] Thomas A.W., Garner L.E., Nevin K.P., Woodard T.L., Franks A.E., Lovley D.R., Sumner J.J., Sund C.J., Bazan G.C. (2013). A lipid membrane intercalating conjugated oligoelectrolyte enables electrode driven succinate production in Shewanella. Energ. Environ. Sci..

[25] Hou H.J., Chen X.F., Thomas A.W., Catania C., Kirchhofer N.D., Garner L.E., Han A., Bazan G.C. (2013). Conjugated Oligoelectrolytes Increase Power Generation in E. coli Microbial Fuel Cells. Adv. Mater..

[26] Gwozdzinska P., Pawlowska R., Milczarek J., Garner L.E., Thomas A.W., Bazan G.C., Chworos A. (2014). Phenylenevinylene conjugated oligoelectrolytes as fluorescent dyes for mammalian cell imaging. Chem. Commun..

[27] Milczarek J., Pawlowska R., Zurawinski R., Lukasik B., Garner L.E., Chworos A. (2017). Fluorescence and confocal imaging of mammalian cells using conjugated oligoelectrolytes with phenylenevinylene core. J. Photochem. Photobiol. B Biol..

[28] Czernek L., Chworos A., Duchler M. (2015). The Uptake of Extracellular Vesicles is Affected by the Differentiation Status of Myeloid Cells. Scand. J. Immunol..

[29] Pu K.Y., Li K., Zhang X.H., Liu B. (2010). Conjugated Oligoelectrolyte Harnessed Polyhedral Oligomeric Silsesquioxane as Light-Up Hybrid Nanodot for Two-Photon Fluorescence Imaging of Cellular Nucleus. Adv. Mater..

[30] Bang H.S., Seo S.Y., Choo D.C., Kim T.W., Lee S.J., Seo J.H., Kim Y.K., Chu C., Ha J. (2009). Effect of doped emitting layer on electrical and optical properties in blue organic light-emitting devices. Thin Solid Films.

[31] Qi Q.J., Wu X.M., Hua Y.L., Hou Q.C., Dong M.S., Mao Z.Y., Yin B., Yin S.G. (2010). Enhancement of performance for blue organic light emitting devices based on double emission layers. Org. Electron..

[32] Kuboshima D., Miyamoto E., Hamasaki K., Nakai N., Inagaki Y., Okada H., Ichiguchi T., Maruo K. (2006). Electrophotographic Photoreceptor and Image Forming Apparatus. Patent.

[33] Schinzel E., Frischkorn H., Roesch G. (1976). 1,2,4-triazolyl (1) Derivatives, Processes for Their Preparation and Their Use as Optical Brightening Agents. Patent.

[34] Sanchez M.I., Martinez-Costas J., Mascarenas J.L., Vazquez M.E. (2014). MitoBlue: A nontoxic and photostable blue-emitting dye that selectively labels functional mitochondria. ACS Chem. Biol..

[35] Carvalho T.O., Carvalho P., Correa J.R., Guido B.C., Medeiros G.A., Eberlin M.N., Coelho S.E., Domingos J.B., Neto B.A.D. (2019). Palladium Catalyst with Task-Specific Ionic Liquid Ligands: Intracellular Reactions and Mitochondrial Imaging with Benzothiadiazole Derivatives. J. Organ. Chem..

[36] Lukasik B., Milczarek J., Pawlowska R., Zurawinski R., Chworos A. (2017). Facile synthesis of fluorescent distyrylnaphthalene derivatives for bioapplications. New J. Chem..

[37] Chworos A., Zurawinski R., Lukasik B., Pawlowska R., Milczarek J. (2018). Pochodne distyrylonaftalenu, sposób ich wytwarzania oraz ich zastosowanie.

[38] Du J., Thomas A.W., Chen X., Garner L.E., Vandenberg C.A., Bazan G.C. (2013). Increased ion conductance across mammalian membranes modified with conjugated oligoelectrolytes. Chem. Commun. (Camb).

[39] Hinks J., Wang Y., Poh W.H., Donose B.C., Thomas A.W., Wuertz S., Loo S.C., Bazan G.C., Kjelleberg S., Mu Y. (2014). Modeling cell membrane perturbation by molecules designed for transmembrane electron transfer. Langmuir.

[40] Kowalska-Baron A., Zurawinski R., Lukasik B., Chworos A., Wysocki S. (2017). Solvent effects on the photophysical properties of distyrylnaphthalene-based conjugated oligoelectrolytes. J. Lumin..

[41] Kowalska-Baron A., Zurawinski R., Lukasik B., Chworos A., Wysocki S. (2018). Theoretical and experimental study on the electronic structure of distyrylnaphthalene-based conjugated oligoelectrolytes. J. Lumin..

[42] Shim S.H., Xia C., Zhong G., Babcock H.P., Vaughan J.C., Huang B., Wang X., Xu C., Bi G.Q., Zhuang X. (2012). Super-resolution fluorescence imaging of organelles in live cells with photoswitchable membrane probes. Proc. Natl. Acad. Sci. USA.

[43] Kwiatek J.M., Owen D.M., Abu-Siniyeh A., Yan P., Loew L.M., Gaus K. (2013). Characterization of a new series of fluorescent probes for imaging membrane order. PLoS ONE.

[44] Guo L., Zhang R., Sun Y., Tian M., Zhang G., Feng R., Li X., Yu X., He X. (2016). Styrylpyridine salts-based red emissive two-photon turn-on probe for imaging the plasma membrane in living cells and tissues. Analyst.

[45] Mukherjee T., Aravintha Siva M., Bajaj K., Soppina V., Kanvah S. (2020). Imaging mitochondria and plasma membrane in live cells using solvatochromic styrylpyridines. J. Photochem. Photobiol. B, Biol..

[46] Heek T., Nikolaus J., Schwarzer R., Fasting C., Welker P., Licha K., Herrmann A., Haag R. (2013). An amphiphilic perylene imido diester for selective cellular imaging. Bioconjug. Chem..

[47] Collot M., Boutant E., Lehmann M., Klymchenko A.S. (2019). BODIPY with Tuned Amphiphilicity as a Fluorogenic Plasma Membrane Probe. Bioconjug. Chem..

