# Supplementary material for: Highly Fluorescent Distyrylnaphthalene Derivatives as a Tool for Visualization of Cellular Membranes
Source: Materials (Basel). 2020 Feb 20;13(4):951. doi: 10.3390/ma13040951 (PMC7078901; doi:10.3390/ma13040951)
Supplement: Supplementary file 1 [file materials-13-00951-s001.pdf]

SUPPLEMENTARY INFORMATION

Highly Fluorescent Distyrylnaphthalene Derivatives as a Tool for Visualization of Cellular Membranes

Justyna Suwara, Beata Lukasik, Remigiusz Zurawinski, Roza Pawlowska*, Arkadiusz Chworos*

Centre of Molecular and Macromolecular Studies, Polish Academy of Sciences, Sienkiewicza 112, 90-363 Lodz, Poland
* Corresponding authors. E-mail addresses: achworos@cbmm.lodz.pl (A.C.), rozapech@cbmm.lodz.pl (R.P.)

1. General remarks

Unless stated otherwise, all air and water sensitive reactions were carried out under an argon atmosphere using freshly distilled dry solvents. All glassware was dried prior to use by heating under vacuum. Dimethyl 2,6-naphthalenedicarboxylate, diethanolamine, morpholine and trimethyl phosphite were purchased from Sigma-Aldrich. Commercial grade reagents and solvents were used without further purification except as indicated below. THF was distilled over Na/benzophenone prior to use. NMR spectra were recorded on Bruker DRX 500 spectrometer. ^{1}H, ^{13}C chemical shifts are reported relative to the residual proton resonance in the deuterated solvents. All chemical shifts (δ) are given in ppm and the coupling constants (*J*) in Hz. Positive ion MALDI mass spectra were recorded on a Voyager-Elite (PerSeptive Biosystems Inc., Framingham, MA, USA) instrument equipped with nitrogen laser (337 nm) in a linear mode at an acceleration voltage of 20 kV and delayed extraction. Melting points are uncorrected.

2,6-Bis[4-(*N,N*-bis{6-[bis(2-hydroxyethyl)amino]hexyl}amino)styryl]naphthalene (DSNN -DEA): A solution of **DSNN-I** (68mg, 0.056mmol) and diethanolamine (0.71g, 6.78mmol) in THF (10mL) was stirred at 70°C for 6h. The solvent and an excess of diethanolamine were evaporated under the reduced pressure. To the residue, saturated aqueous $NaHCO_3$ solution (5mL) was added and the mixture was extracted with CH_2Cl_2 (3 x 20mL). The combined organic extracts were washed with water and dried over Na_2SO_4. Evaporation of the solvent afforded **DSNN-DEA** (62mg, 98%) as a yellow-green solid. Mp 86-87 ^{o}C; ^{1}H NMR (500 MHz, $CDCl_3$) δ 7.74 (d, *J* = 8.3 Hz, 2 H, $C_{Ar}H$), 7.73 (s, 2 H, $C_{Ar}H$), 7.68 (d, *J* = 8.7 Hz, 2 H,

C_{Ar}*H*), 7.41 (d, *J* = 8.5 Hz, 4 H, C_{Ar}*H*), 7.14 (d, *J* = 16.2 Hz, 2 H, C*H*=CH), 7.02 (d, *J* = 16.1 Hz, 2 H, C*H*=CH), 6.63 (d, *J* = 8.6 Hz, 4 H, C_{Ar}*H*), 3.62 (t, *J* = 5.3 Hz, 16 H, OCH_2), 3.29 (t, *J* = 7.2 Hz, 8 H, C_{Ar}NCH_2), 2.65 (t, *J* = 5.2 Hz, 24 H, NC$H_2$$CH_2$O and O*H*), 2.53 (t, *J* = 7.3 Hz, 8 H, $(HOCH_2CH_2)_2$NCH_2), 1.61 (br s, 8 H, CH_2), 1.49 (br s, 8 H, CH_2), 1.35 (br s, 16 H, CH_2); ^{13}C NMR (126 MHz, $CDCl_3$) δ 147.76 (2 C, C_{Ar}N), 135.32 (2 C, C_{Ar}), 132.94 (2 C, C_{Ar}), 128.82 (2 C, *C*H=CH), 127.98 (2 C, C_{Ar}H), 127.77 (4 C, C_{Ar}H), 125.21 (2 C, C_{Ar}H), 124.76 (2 C, C_{Ar}), 123.88 (2 C, C_{Ar}H), 123.83 (2 C, *C*H=CH), 111.79 (4 C, C_{Ar}H), 59.53 (8 C, OCH_2), 56.09 (8 C, NC$H_2$$CH_2$OH), 54.71 (4 C, $(HOCH_2CH_2)_2$NCH_2), 51.12 (4 C, C_{Ar}NCH_2), 27.39 (4 C, CH_2), 27.13 (8 C, CH_2), 26.99 (4 C, CH_2); MALDI MS: $[M+H]^+$ 1111.7; Anal. calcd. for $C_{66}H_{106}N_6O_8$: C, 71.31; H, 9.61. Found: C, 71.09; H, 9.73.

2. NMR spectra

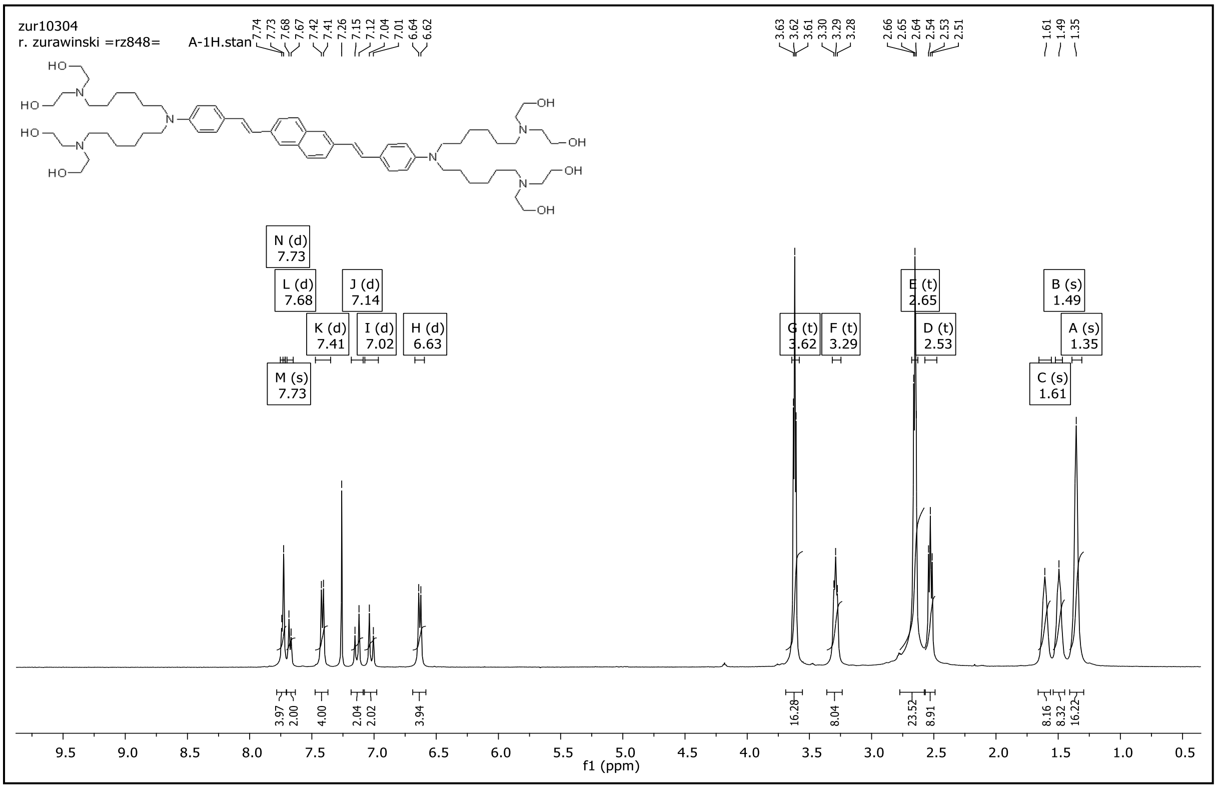

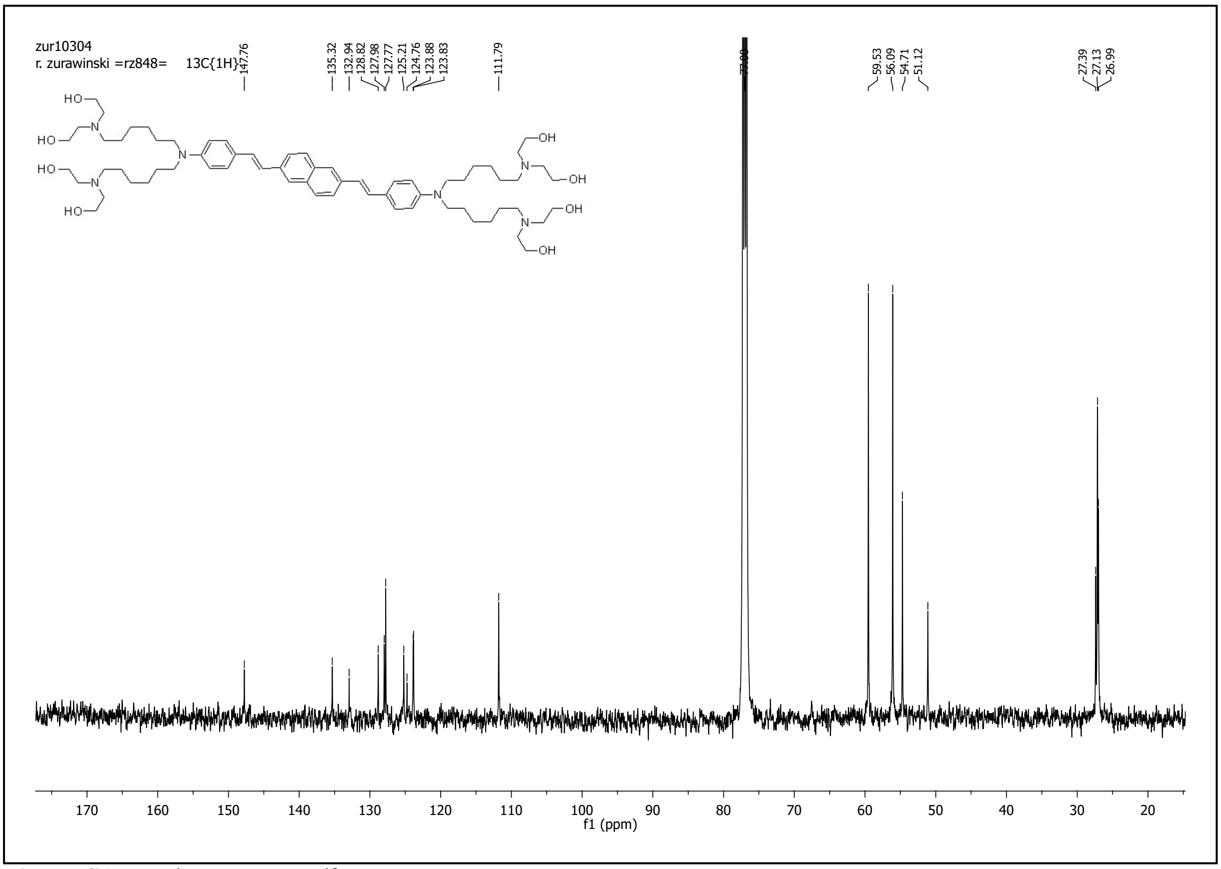

Figure S1. The ¹H-NMR and ¹³C-NMR spectra of DSNN-DEA derivative.

3. Cytotoxicity results

Table S1. In vitro cytotoxicity assay results (% living cells) performed on various cell lines after 72h of treatment with DSNN-compounds in 1µM, 5µM and 10µM. The results represent the mean ± standard error.

		MDM	NIH/3T3	293T	Fibroblasts	HCT116	HeLa	K562	HUVEC
DSNN-NMe_3^+	1µM	60.0±3.7	80.1±3.5	90.8±3.9	93.1±7.6	104.2±7.7	*91.5±2.8	107.5±2.9	88.8±5.2
	5µM	19.2±1.0	43.1±1.7	50.7±4.2	46.0±5.2	59.4±1.9	*59.9±2.0	43.5±1.6	79.1±4.9
	10µM	17.0±1.4	41.7±1.3	46.1±3.2	47.7±3.1	43.6±3.0	*47.8±2.5	37.1±0.1	64.7±4.7
DSNN-P	1µM	76.5±4.3	100.5±2.5	99.9±3.5	86.3±4.2	103.4±8.6	*92.5±4.0	91.2±4.2	88.2±2.4
	5µM	74.5±5.8	100.5±1.4	100.9±4.4	97.5±4.0	112.0±6.7	*91.1±1.7	90.5±1.1	73.4±3.2
	10µM	76.0±4.2	100.1±2.4	103.3±2.5	97.5±6.1	104.7±7.7	*95.8±1.9	93.2±2.2	67.6±4.1
DSNN-Mor	1µM	82.5±7.1	107.1±2.9	97.7±2.1	97.3±3.0	108.6±5.8	*96.6±3.7	94.5±0.9	89.5±5.5
	5µM	69.5±6.9	109.7±3.5	107.7±2.6	91.3±2.6	104.2±2.9	*99.3±4.1	86.4±4.0	66.9±0.5
	10µM	82.8±6.5	103.0±4.0	96.0±3.3	104.4±3.7	115.0±5.3	*90.1±3.5	94.9±3.3	70.2±4.7
DSNN-DEA	1µM	70.6±5.4	80.7±5.3	97.0±1.5	90.8±5.7	97.8±1.9	85.3±4.5	85.2±4.5	83.3±3.2
	5µM	22.7±5.8	90.4±1.1	56.7±3.6	51.5±4.4	35.3±3.6	36.0±3.4	38.8±0.8	66.3±0.8
	10µM	20.4±1.2	27.7±2.2	41.9±3.4	8.5±2.0	24.7±3.1	30.4±2.9	37.1±0.3	58.4±3.5
DSNN-POK	1µM	101.5±3.4	100.8±5.1	97.8±1.3	84.6±2.4	97.8±2.7	*83.7±3.1	92.7±4.9	97.8±3.2
	5µM	94.3±4.8	98.9±2.7	92.6±2.7	81.6±3.5	82.2±2.1	*86.0±2.1	95.5±2.3	83.8±3.7
	10µM	86.6±4.3	84.9±4.5	100.9±1.6	103.9±4.2	88.9±3.3	*84.7±3.4	84.0±3.2	67.8±5.3
DSNN-NH_2	1µM	73.3±4.7	99.2±3.4	98.5±1.3	76.8±2.2	89.1±2.5	*87.8±3.1	96.8±4.8	93.5±4.8
	5µM	31.2±3.4	59.3±3.1	90.7±4.9	62.9±3.5	82.9±2.6	*87.2±1.2	78.8±9.7	99.0±2.9
	10µM	32.7±5.9	47.4±1.9	93.9±2.0	62.8±2.7	80.6±4.4	*83.5±2.3	53.4±3.5	93.7±4.6
DSNN-Py^+	1µM	40.4±1.9	77.1±1.4	89.9±2.9	59.7±4.9	95.6±6.4	*87.1±3.8	107.3±2.1	89.8±2.3
	5µM	19.6±1.9	58.5±2.0	68.2±9.4	37.9±2.6	42.3±0.9	*75.5±3.4	46.0±1.0	95.9±2.4
	10µM	17.3±4.4	46.4±2.9	72.2±7.6	41.2±9.9	39.9±2.3	*68.9±3.6	38.9±0.8	99.0±3.4

4. Absorption and emission spectra of DSNN-DEA derivative

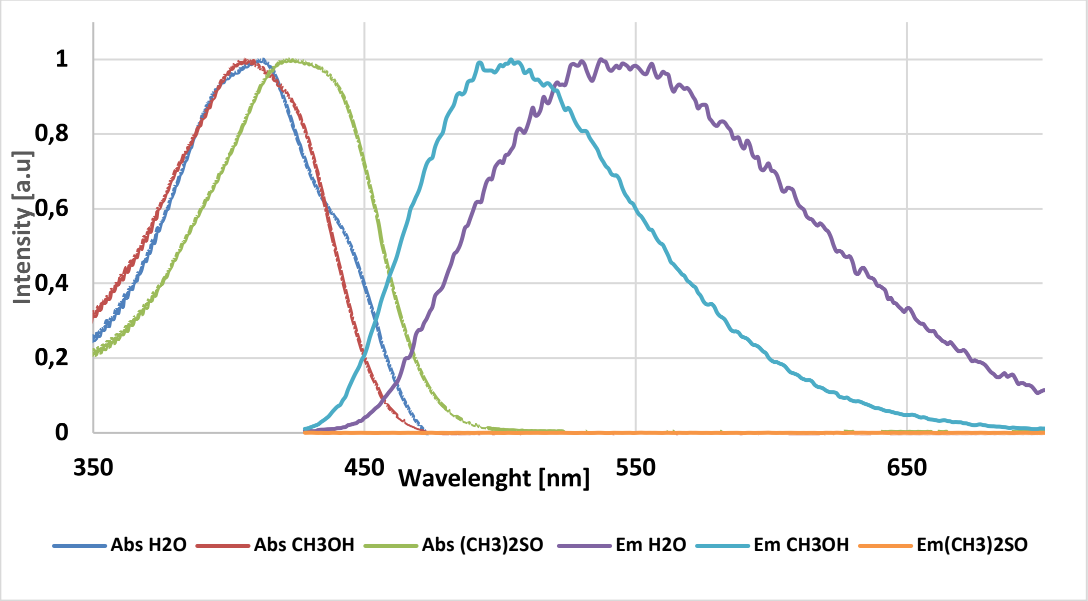

Figure S2. Normalized excitation and emission spectra (PL) of DSNN-DEA derivative in various solvents: water (excitation – dark blue, emission - violet), MeOH (excitation - red, emission - blue), DMSO (excitation - green, emission - orange).

5. Fluorescence and confocal microscopy images

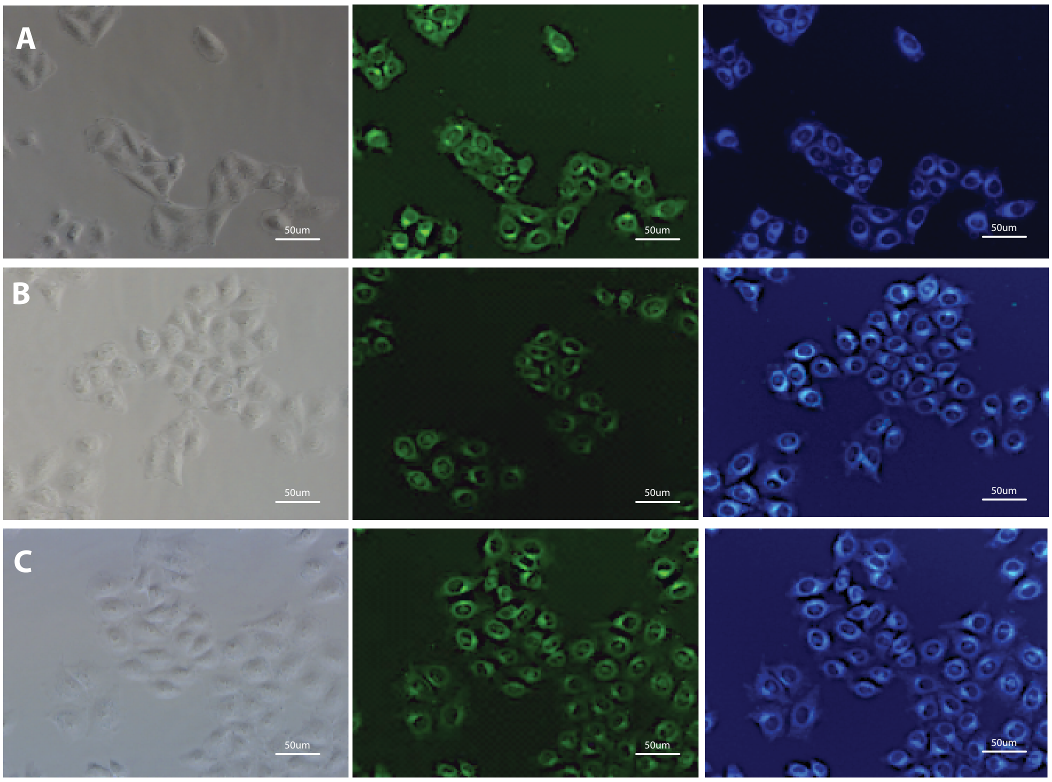

Figure S3. Fluorescence microscopy images of live HeLa cells after 24-hours incubation with DSNN-NMe$_3^+$ in various culture media. A) HBSS, B) RPMI1640, C) complete medium (RPMI1640, 10% FBS, antibiotics), collected at 40x magnification. Left panel - phase contrasts, middle panel – B2A filter (400ms), right panel – UV-2A filter (300ms). All images are 400x320μm

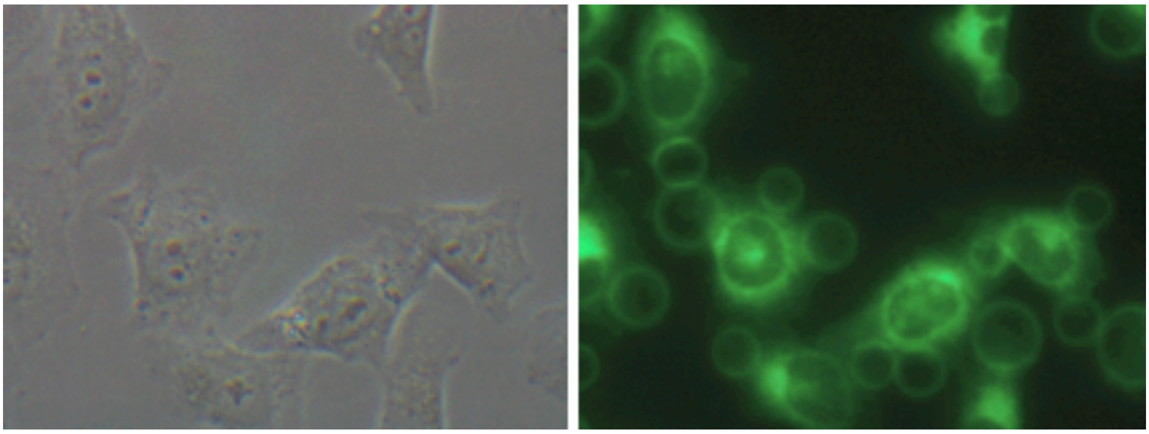

Figure S4. Fluorescence microscopy images of HeLa cells incubated 24h with 1mM DSSN, next incubated 2,5h with 5mM staurosporine. Left panel – phase contrast, right panel – B2A filter (1s).

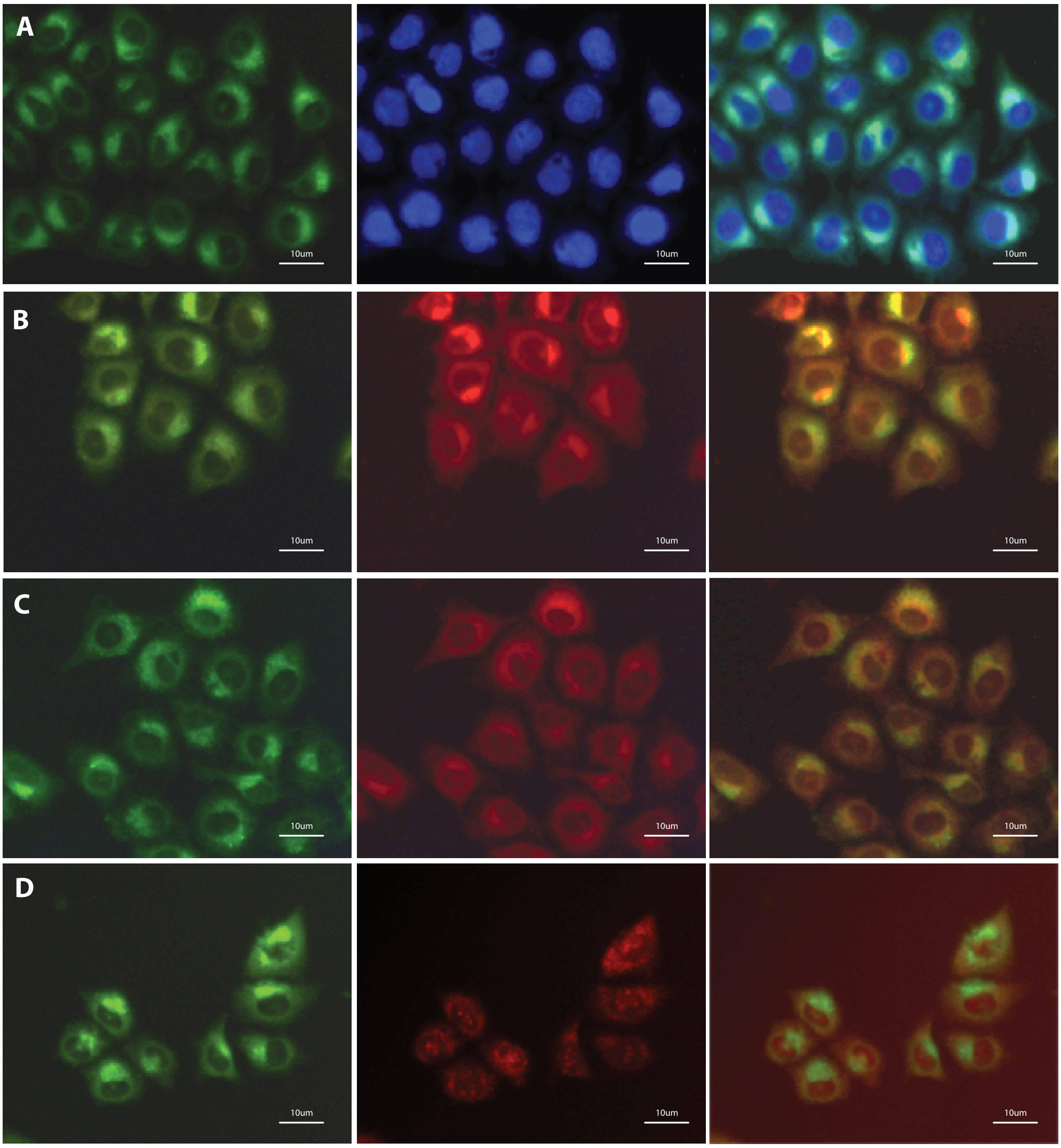

Figure S5. Fluorescence microscopy images of fixed HeLa cells after 24-hours incubation with 1μM DSNN-DEA at 1μM concentration, co-labeled 2-hours with A) 5μg/ml DAPI; B) 5μM BODIPY® TR; C) 1μM ErTracker; D) 0.1 μM MitoTracker Orange, collected at 60x (A) and 40x (B-D) magnification. Left panel - B2A filter (exposure time - A 3s, B 1s, C 3s, D 1s); middle panel – DAPI filter (A 1s) or Texas red filter (B 3s, C 3s, D 3s); right panel – merged of left and middle panels. All images are 80x65μm

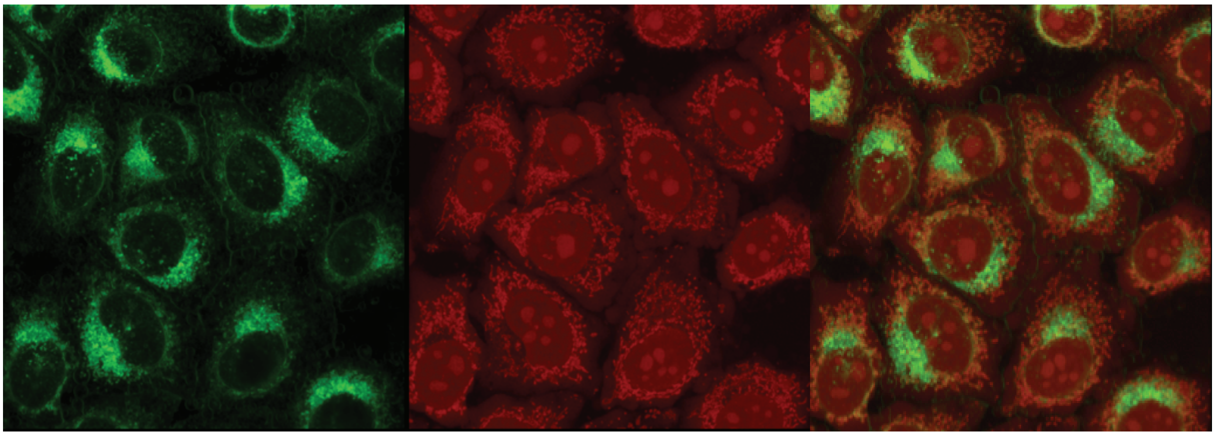

Figure S6. Confocal microscopy images of fixed HeLa cells after 24-hours incubation with 1μM DSNN-DEA at 1μM concentration, co-labeled 2-hours with 0.1μM MitoTracker Orange. Left panel - green fluorescence of tested compounds, middle panel – red fluorescence of MitoTracker, right panel – merged of left and middle panels. All images are 35x35μm.

5. FACS data

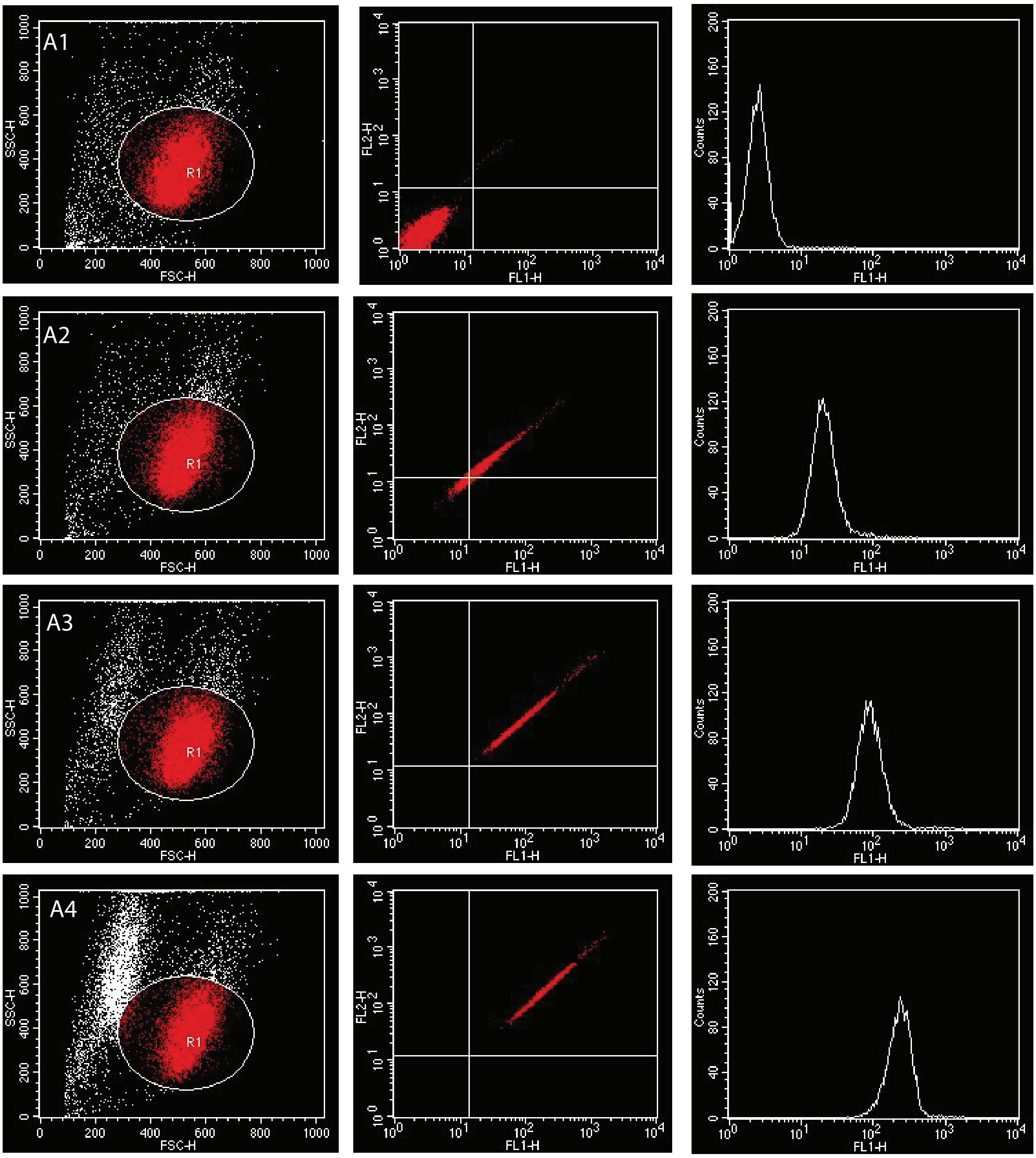

Figure S7 - A. K562 cells incubated1h with 1, 5 i 10μM DSNN-NMe3^{+}

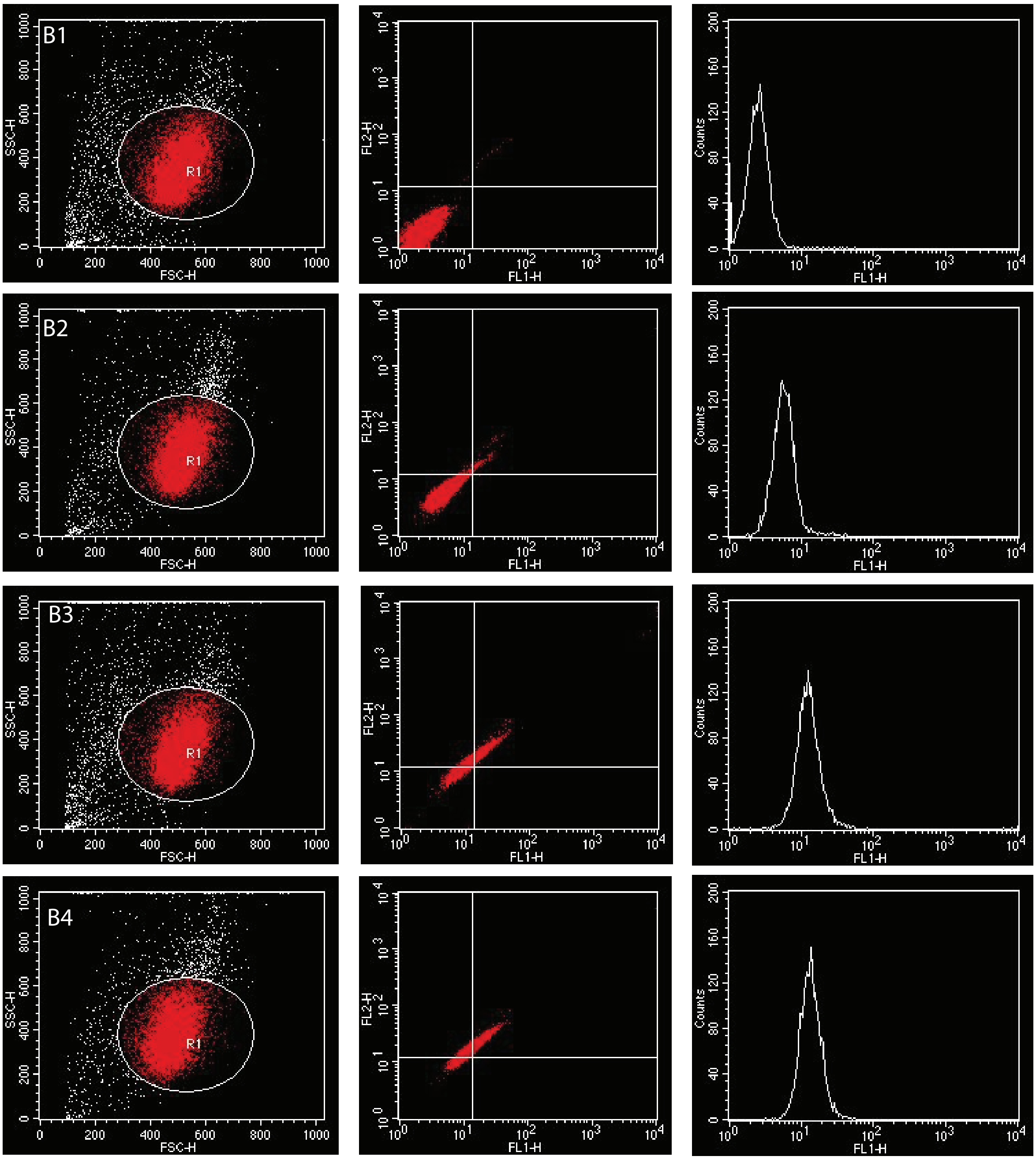

Figure S7 - B. K562 cells incubated 1h with 1, 5 i 10µM DSNN-P.

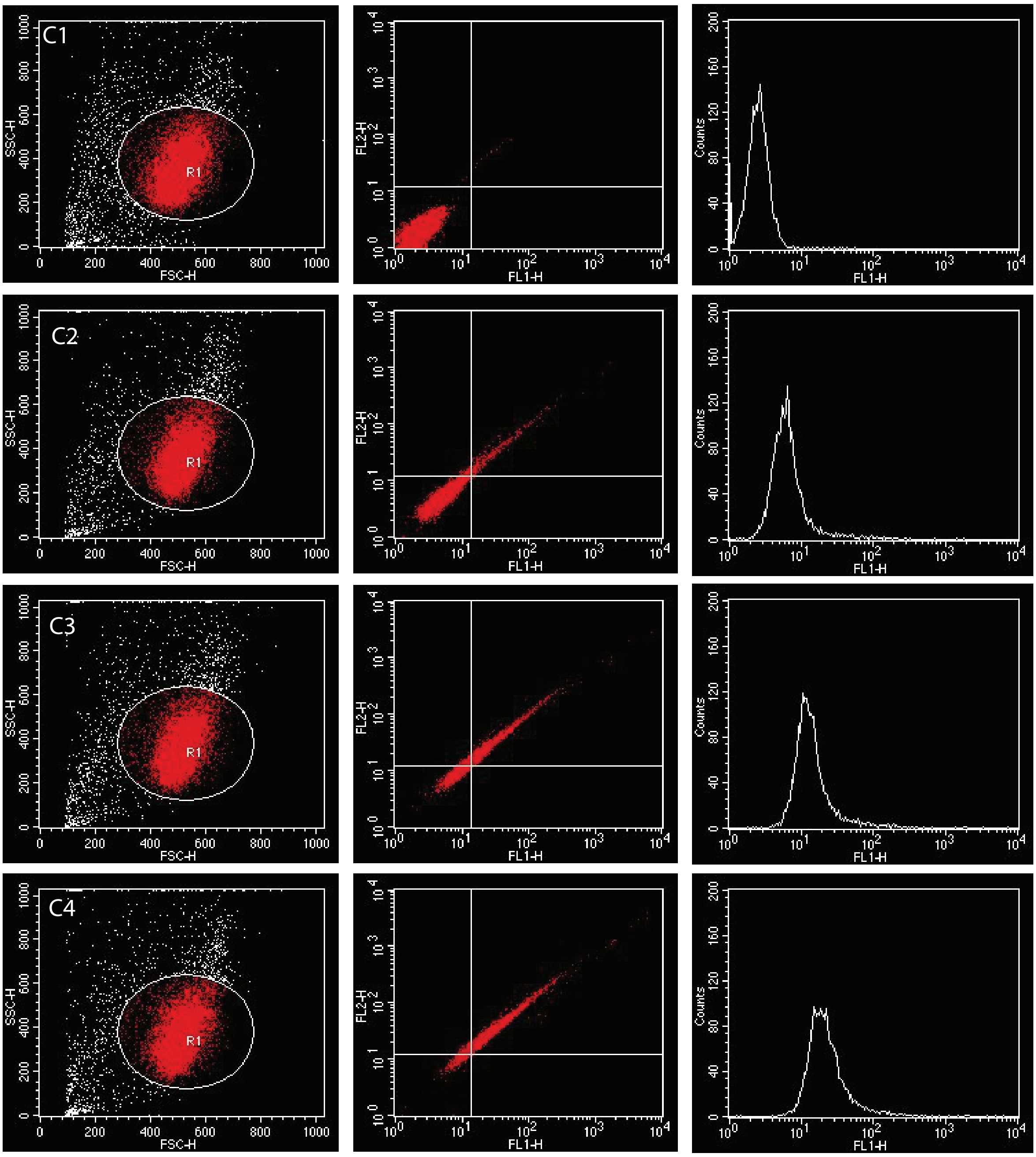

Figure S7 - C. K562 cells incubated1h with 1, 5 i 10μM DSNN-Mor.

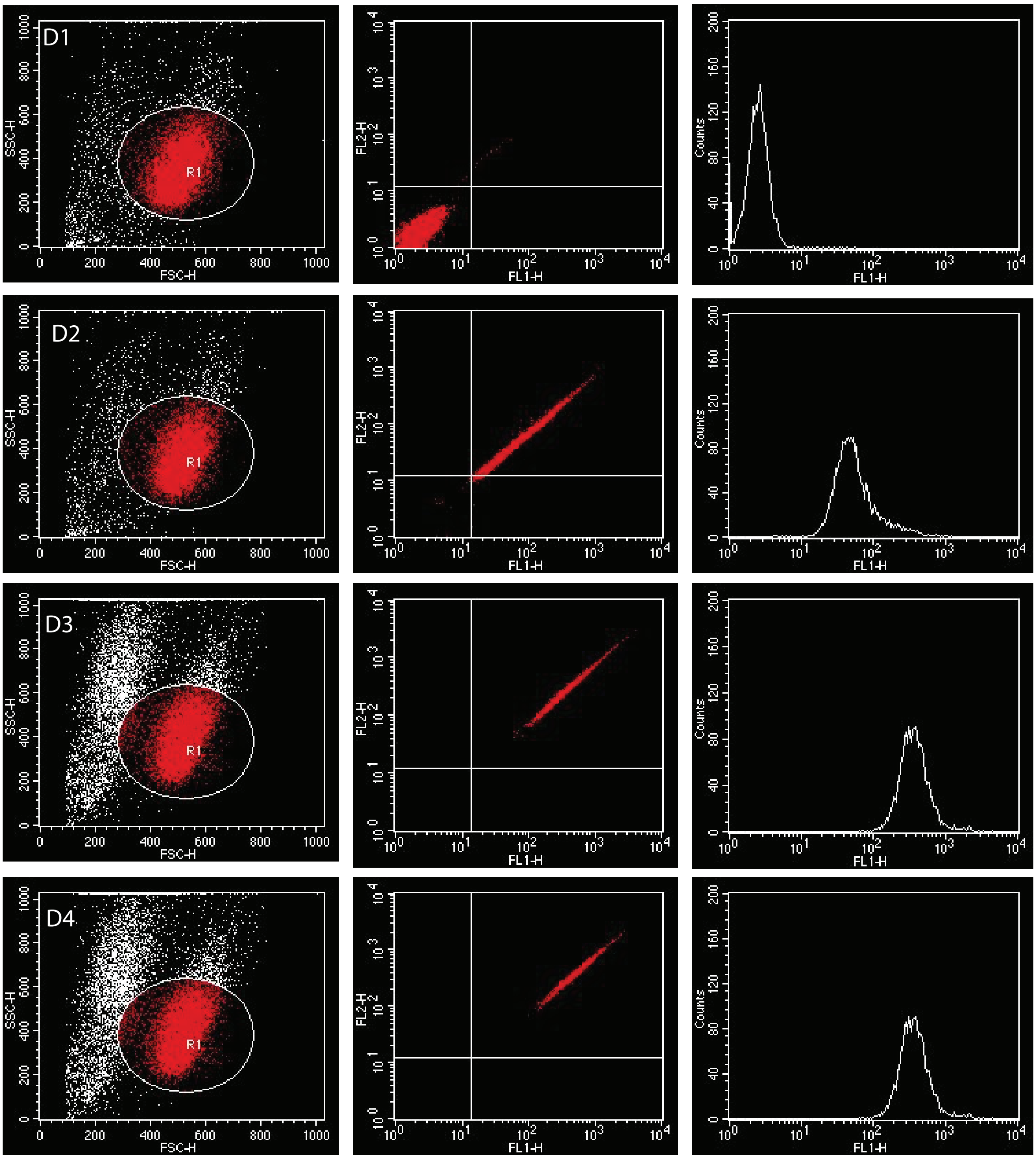

Figure S7 - D. K562 cells incubated1h with 1, 5 i 10µM DSNN_DEA.

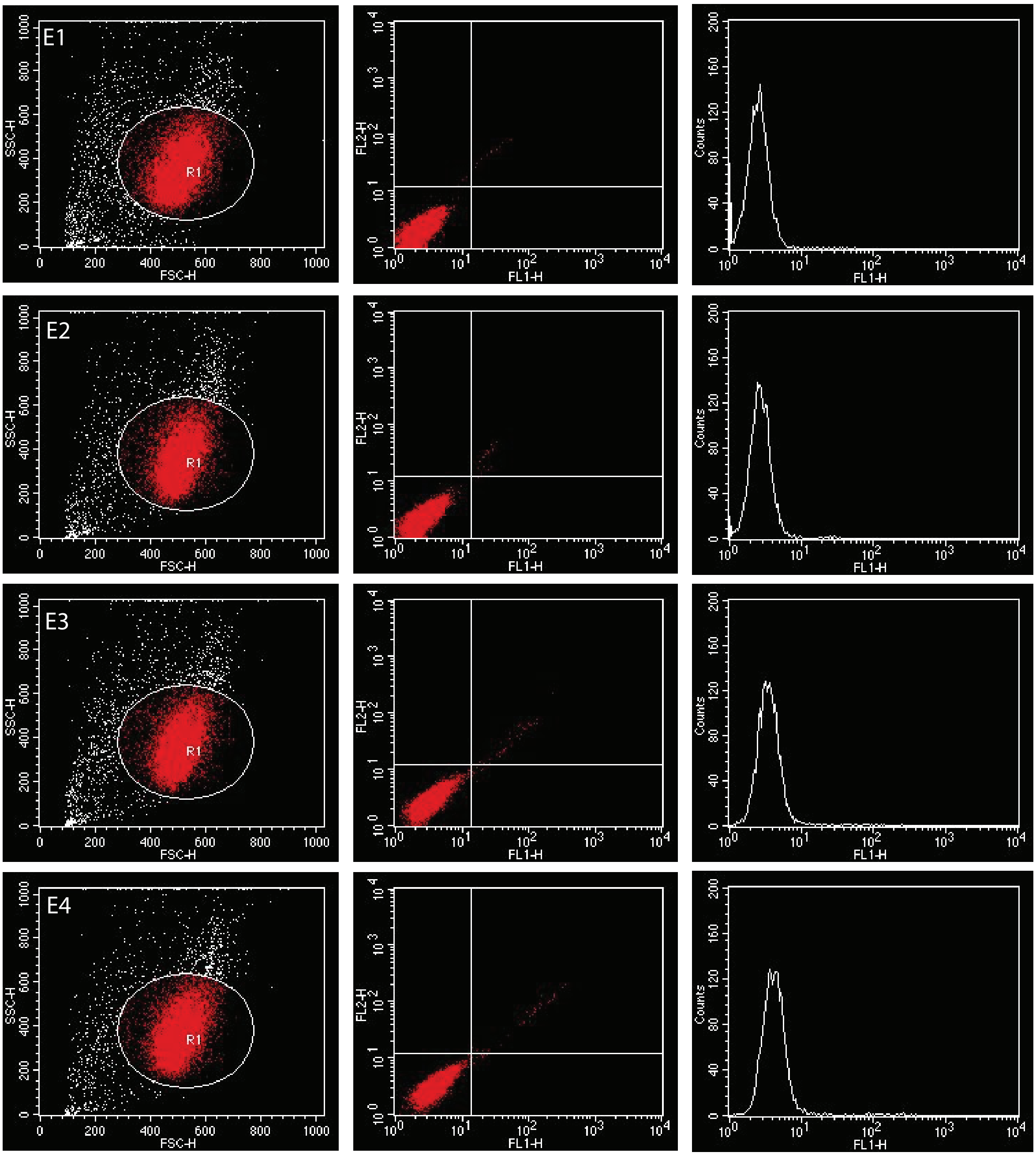

Figure S7 - E. K562 cells incubated1h with 1, 5 i 10μM DSNN-POK.

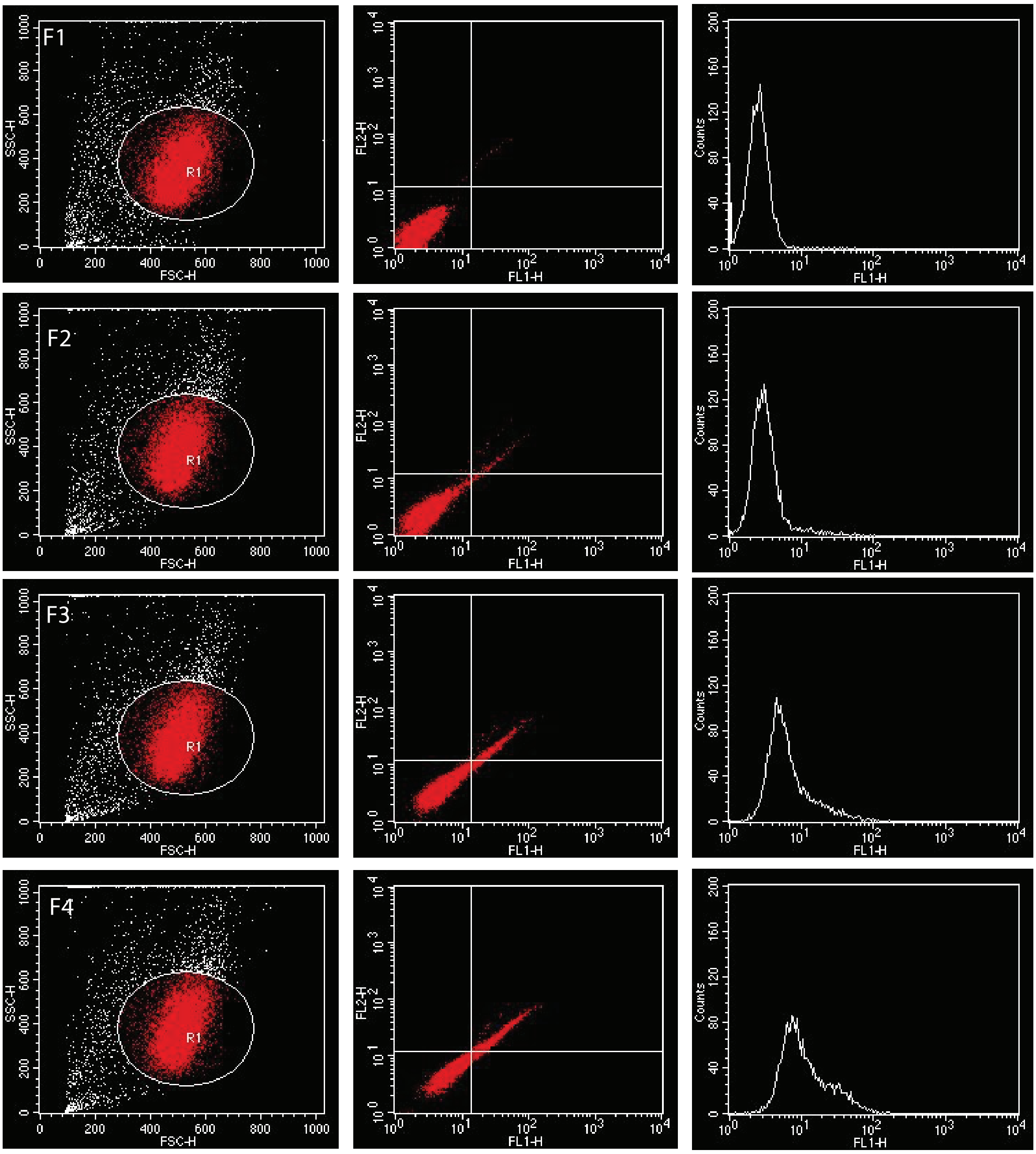

Figure S7 - F. K562 cells incubated1h with 1, 5 i 10µM DSNN-NH_2.

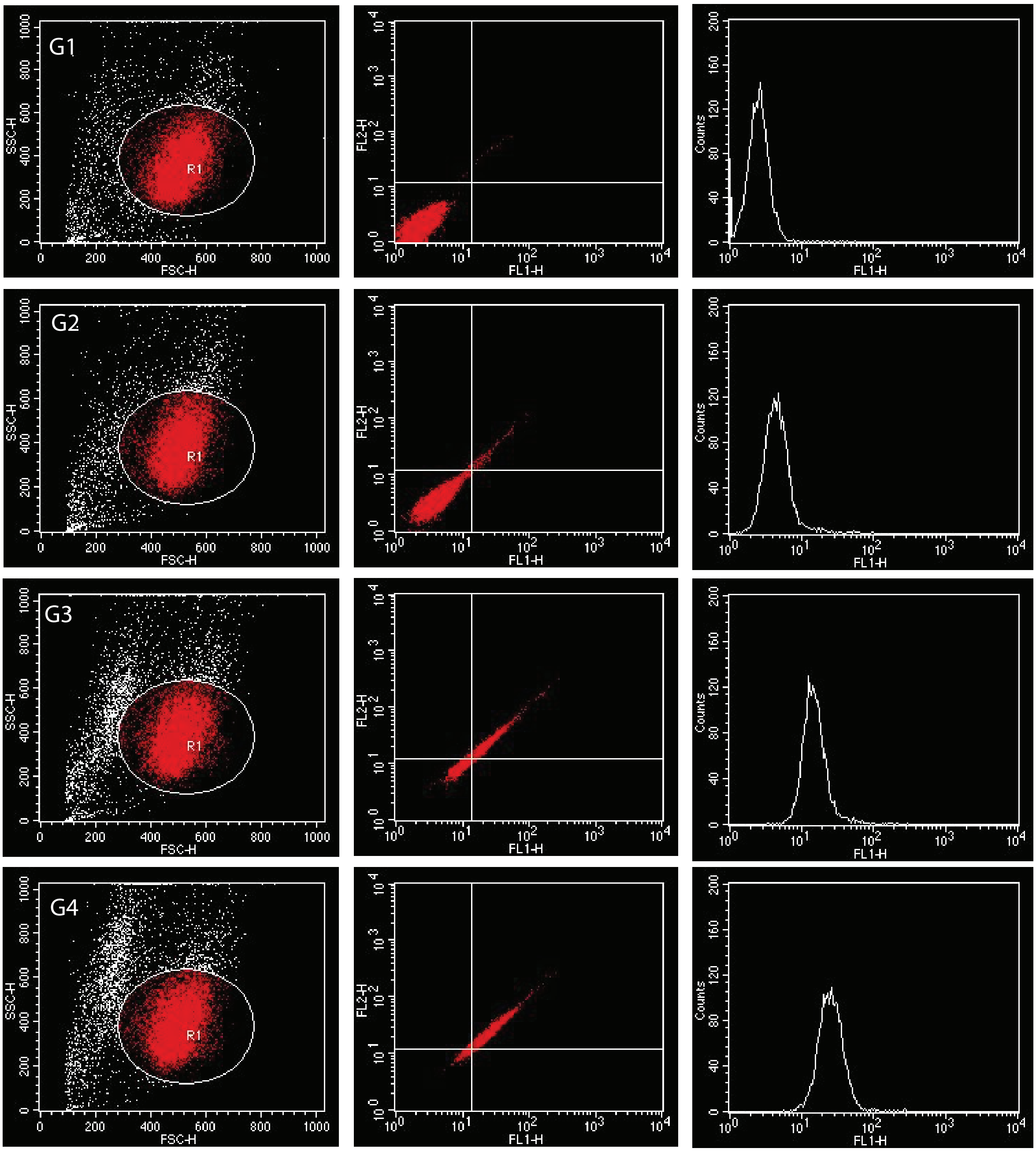

Figure S7 - G. K562 cells incubated1h with 1, 5 i 10µM DSNN-Py⁺

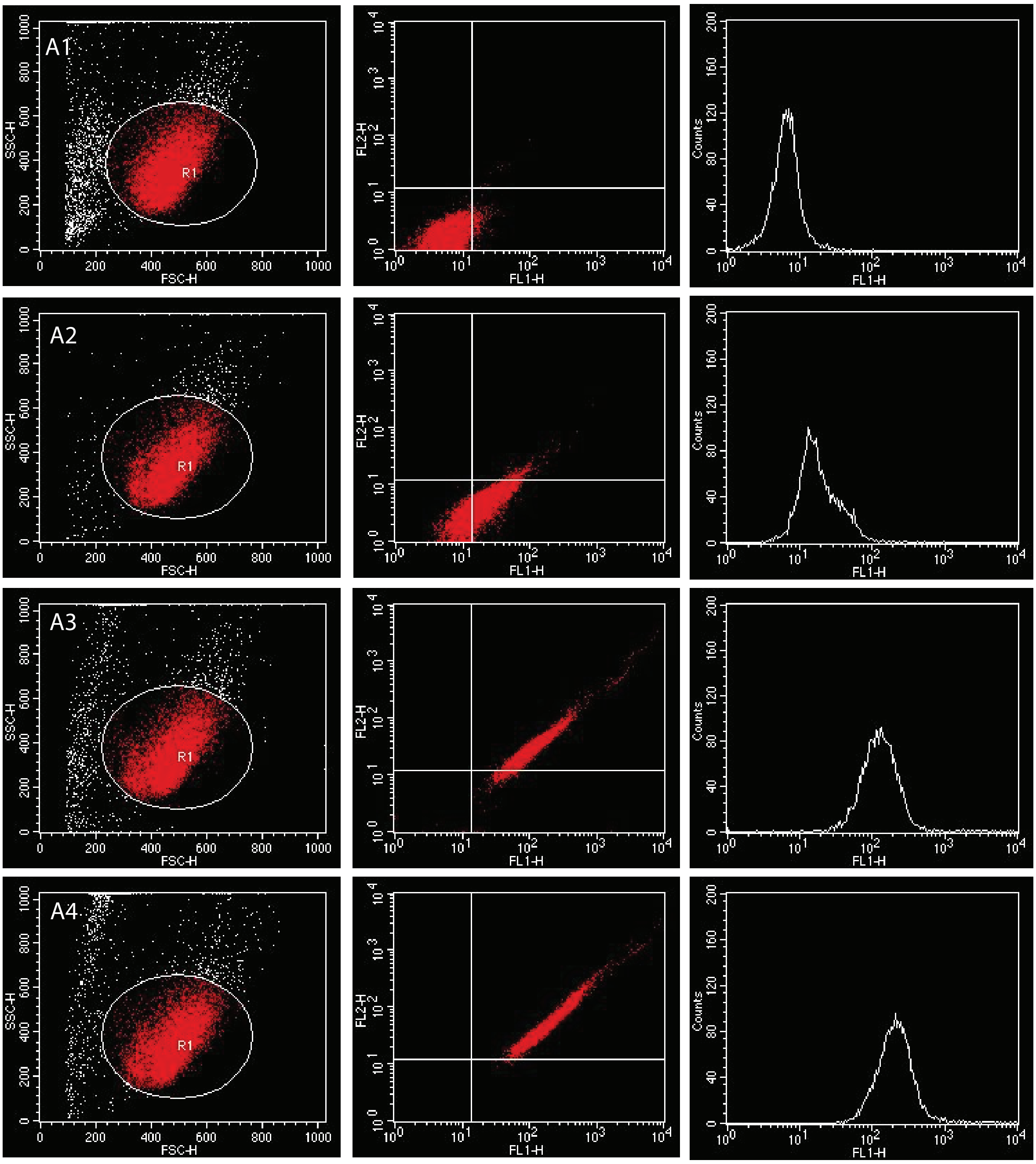

Figure S8 - A. MOLT4 cells incubated1h with 1, 5 i 10μM DSNN-NMe_3^+.

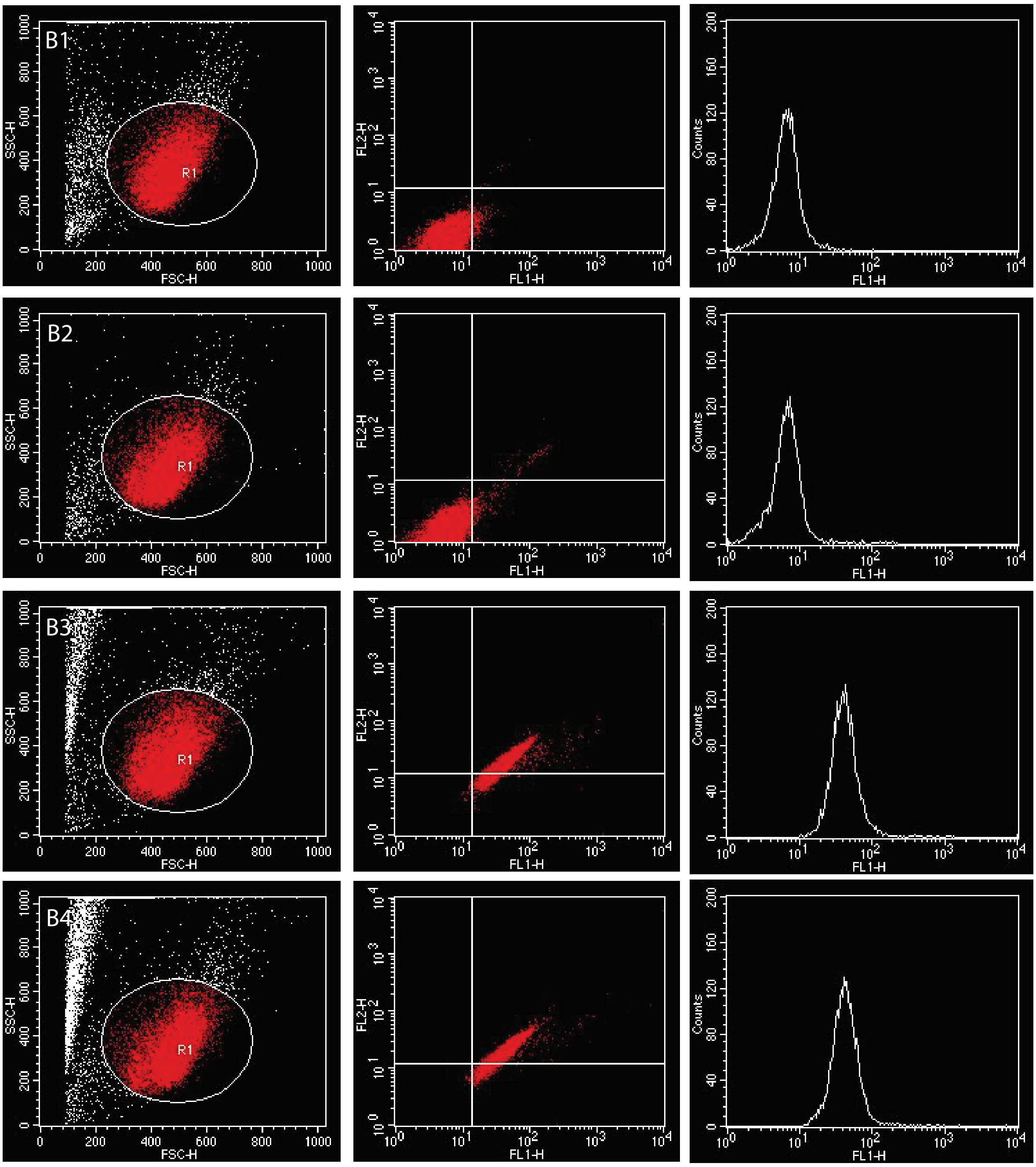

Figure S8 - B. MOLT4 cells incubated1h with 1, 5 i 10µM DSNN-P.

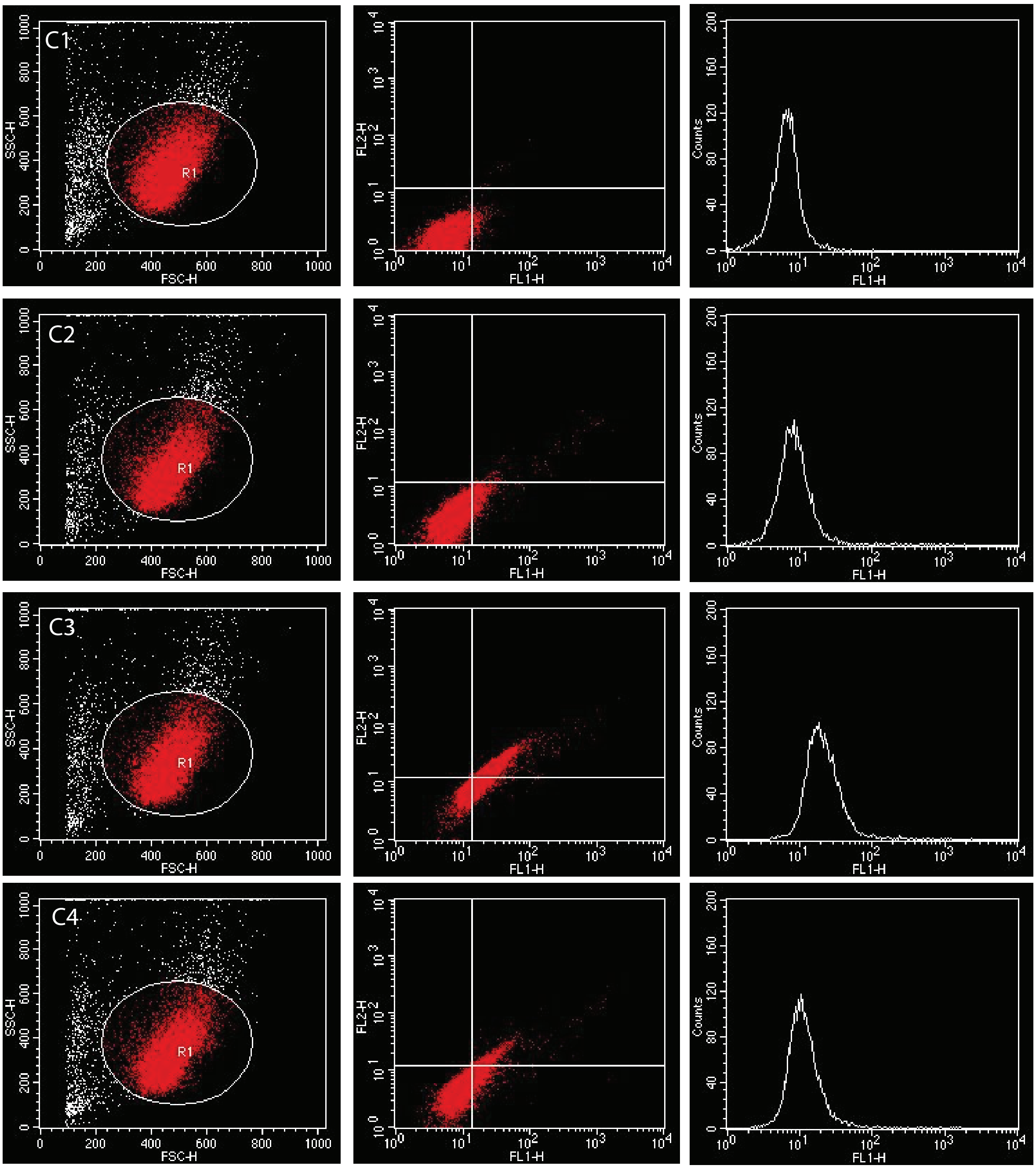

Figure S8 - C. MOLT4 cells incubated1h with 1, 5 i 10μM DSNN-Mor.

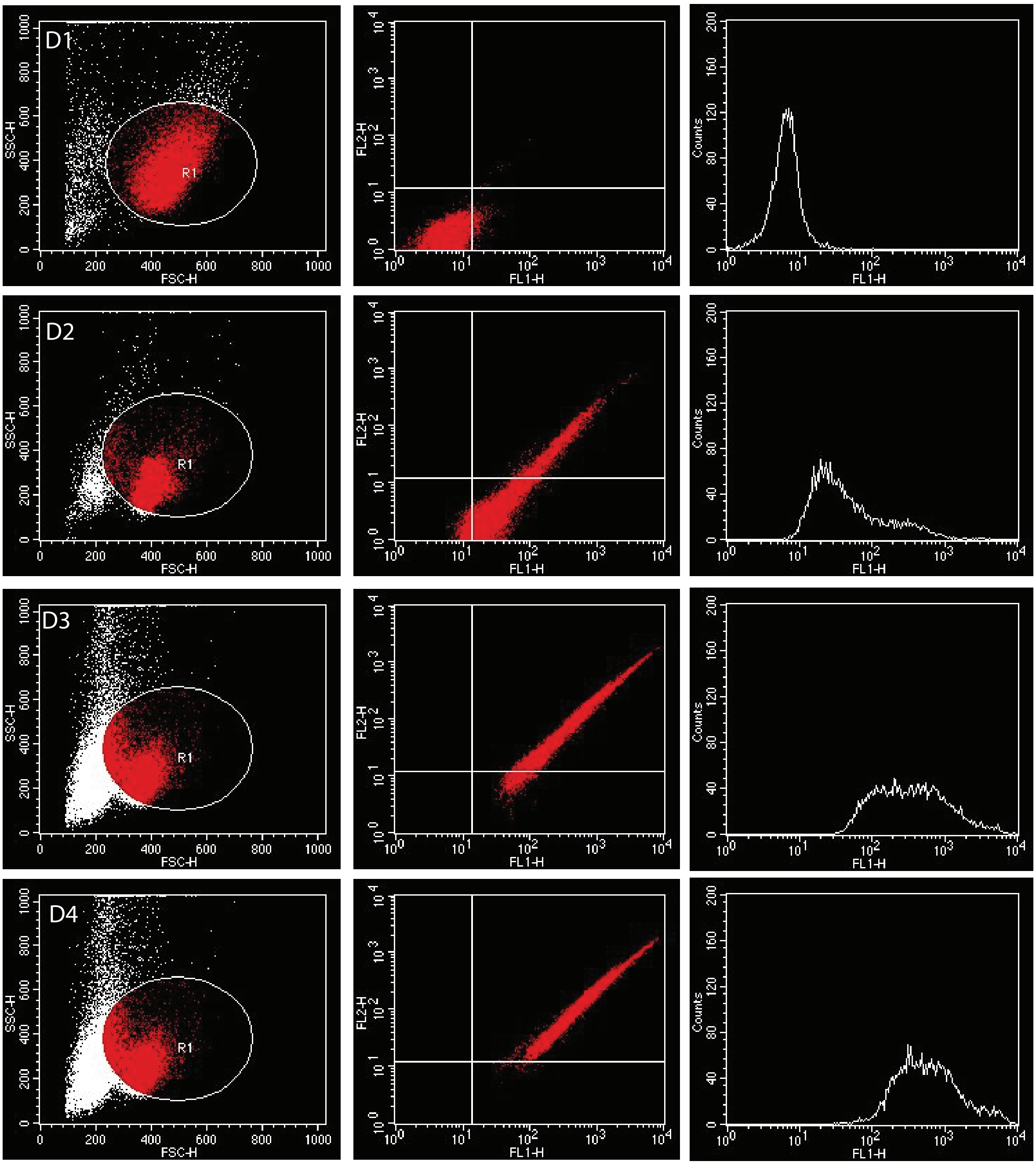

Figure S8_D. MOLT4 cells incubated1h with 1, 5 i 10μM DSNN_DEA.

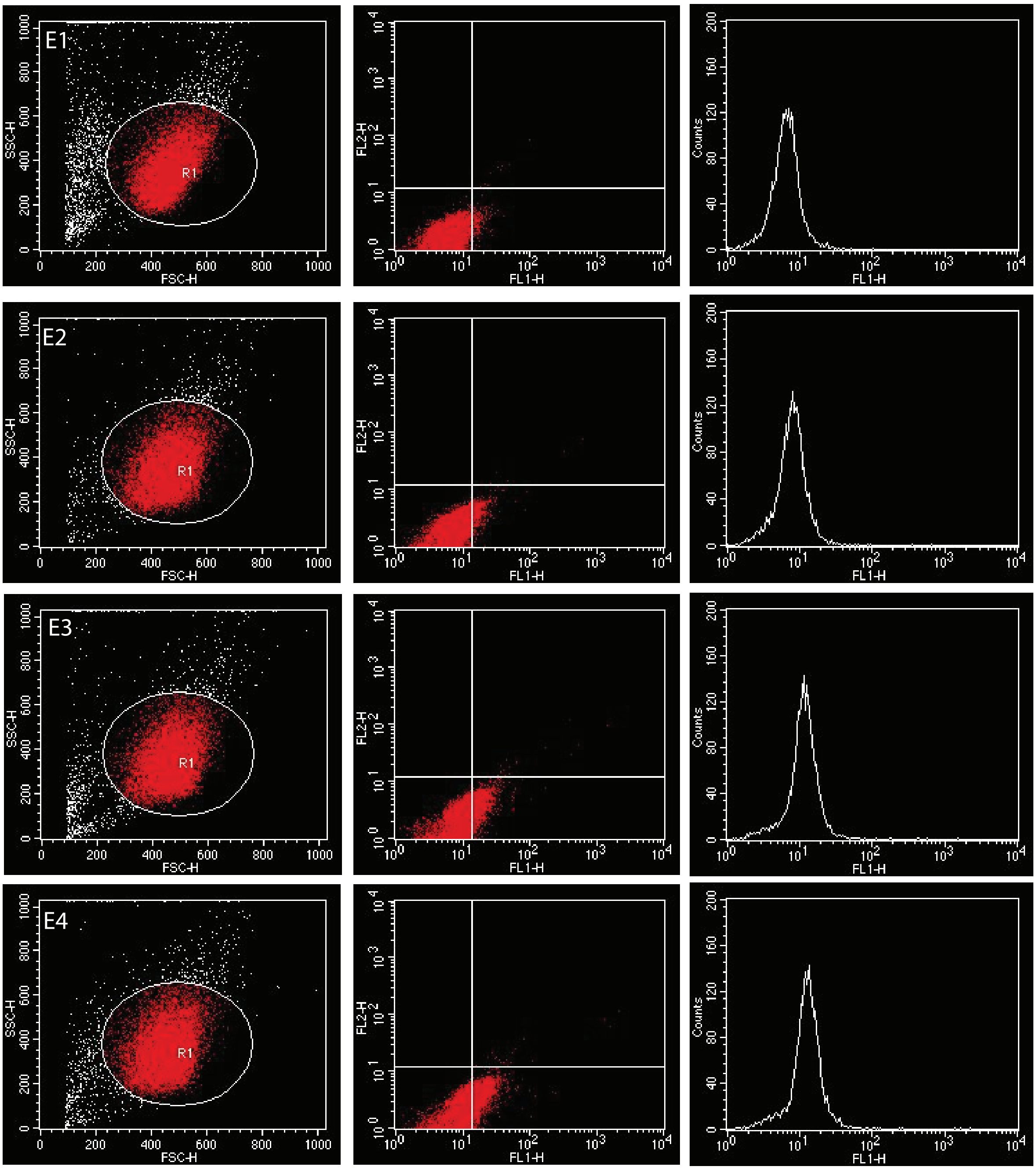

Figure S8 - E. MOLT4 cells incubated1h with 1, 5 i 10μM DSNN-POK.

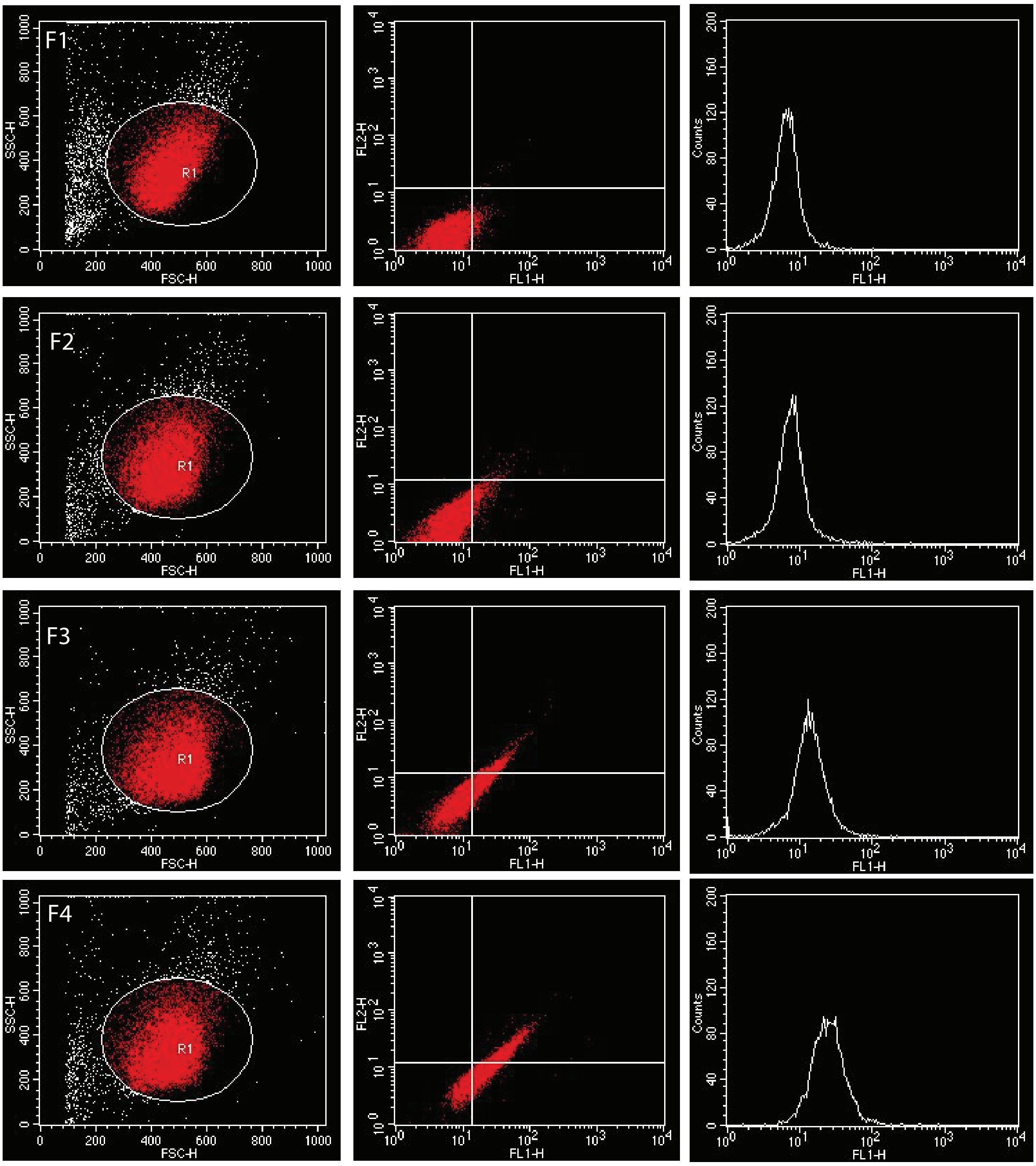

Figure S8 - F. MOLT4 cells incubated1h with 1, 5 i 10μM DSNN-NH_2.

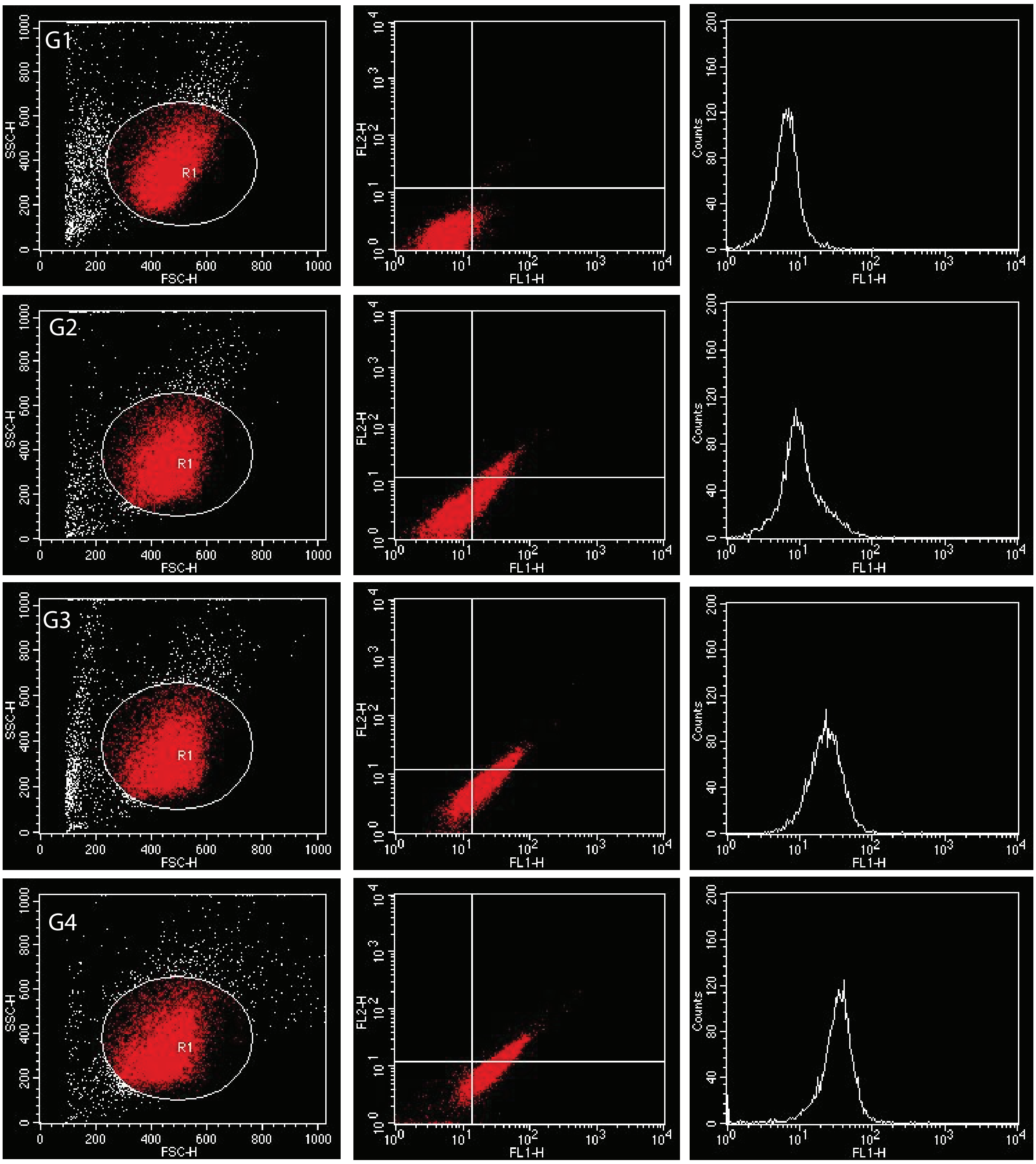

Figure S8 - G. MOLT4 cells incubated1h with 1, 5 i 10μM DSNN-Py$^+$
